# Genetic Determinants of Fatty Acid Composition in Subcutaneous and Visceral Adipose Tissue

**DOI:** 10.1002/oby.70045

**Published:** 2025-09-29

**Authors:** Altayeb Ahmed, Afreen Naz, Marjola Thanaj, Elena P. Sorokin, Brandon Whitcher, Jimmy D. Bell, E. Louise Thomas, Madeleine Cule, Hanieh Yaghootkar

**Affiliations:** ^1^ School of Natural Sciences, College of Health and Science University of Lincoln, Joseph Banks Laboratories Lincoln UK; ^2^ School of Human Development and Health, Faculty of Medicine University of Southampton Southampton UK; ^3^ Research Centre for Optimal Health, School of Life Sciences University of Westminster London UK; ^4^ Calico Life Sciences LLC South San Francisco California USA

**Keywords:** cardiometabolic disease, fatty acids, MRI scan, *SCD1*, visceral adipose tissue

## Abstract

**Objective:**

Fatty acids in adipose tissue are key structural and metabolic regulators of cardiometabolic health, but the genetic architecture governing depot‐specific composition in subcutaneous (SAT) and visceral adipose tissue (VAT) is not well defined.

**Methods:**

We used MRI‐derived estimates of fatty acid composition in SAT and VAT from 33,583 UK Biobank participants to perform genome‐wide association studies. Functional annotation, fine mapping, colocalization, and expression QTL analyses were conducted to prioritize likely causal variants and explore mechanisms.

**Results:**

We identified six loci associated with adipose tissue fatty acid composition, including both shared (*PKD2L1*, *INSIG1*) and depot‐specific associations (*LEKR1* and *KLF14* for SAT; *CDCA2* for VAT). The strongest association, rs603424‐G (near *PKD2L1*), was linked to higher monounsaturated and polyunsaturated fatty acids, lower saturated fatty acids, and increased *SCD1* expression in SAT and VAT, suggesting a role in desaturation and lipid remodeling. Several loci were linked to cardiometabolic outcomes including type 2 diabetes, hypertension, and cholelithiasis, with functional evidence supporting gene–diet interactions at the *PKD2L1* locus.

**Conclusions:**

Our findings uncover genetic determinants of human adipose tissue fatty acid composition, highlight depot‐specific regulation, and point to *SCD1* as a potential metabolic regulator. These results deepen understanding of lipid metabolism and its links to cardiometabolic risk.

**Study Importance**

- High‐resolution MRI used to measure fatty acid composition in SAT and VAT.
- Six genetic loci identified for adipose tissue fatty acid composition traits.
- rs603424‐G linked to *SCD1* expression, driving desaturation and lipid change.
- Genetic loci show distinct associations with cardiometabolic disease risks.
- Depot‐specific effects reveal unique genetic influences in SAT and VAT.

## 1 Introduction

Fatty acids are key structural and metabolic components of adipose tissue, influencing energy storage, membrane composition, and signaling pathways that affect cardiometabolic health [1, 2]. Dietary intake of different fatty acid subtypes—saturated fatty acids (SFAs), monounsaturated fatty acids (MUFAs), and polyunsaturated fatty acids (PUFAs)—has long been thought to influence the risk of type 2 diabetes, cardiovascular disease, and other chronic conditions. For instance, UK dietary guidelines recommend limiting SFA intake to less than 11% of total energy while encouraging the consumption of MUFAs and PUFAs to improve cardiovascular outcomes [3]. However, these general recommendations have been challenged by emerging evidence suggesting that the relationship between dietary fats and disease risk may depend on metabolic context and genetic background [4].

Much of our understanding of fatty acid metabolism and its links to disease has come from studies of circulating fatty acids. Observational and genetic evidence has shown that higher plasma SFAs are associated with increased risk of coronary heart disease, whereas omega‐3 PUFAs are often protective [4, 5, 6]. Mendelian randomization (MR) studies and genetic studies have implicated genetically predicted fatty acid traits—including desaturase activities (e.g., *FADS1*, *FADS2*)—in the pathogenesis of cardiometabolic conditions [7, 8, 9]. For example, higher genetically determined delta‐6‐desaturase (*D6D*) activity has been linked to increased cardiovascular risk, likely through pro‐inflammatory PUFA metabolites [10].

However, blood fatty acid levels represent only one dimension of lipid metabolism and are influenced by both short‐term dietary intake and endogenous regulation. Adipose tissue serves as a long‐term reservoir for fatty acids and may reflect cumulative dietary and metabolic influences. Importantly, the composition of fatty acids stored in adipose tissue differs between depots and may play distinct roles in disease. Subcutaneous adipose tissue (SAT) and visceral adipose tissue (VAT) present different metabolic profiles. VAT is more lipolytically active and more strongly associated with insulin resistance, inflammation, and adverse cardiometabolic outcomes [11]. Previous studies using tissue biopsies have shown that fatty acid profiles in adipose tissue differ by depot and correlate with metabolic risk factors, but such studies have been limited in scale and scope [12, 13].

To address this gap, we used MRI‐derived estimates of fatty acid composition in SAT and VAT from 33,583 UK Biobank participants to investigate the genetic architecture of depot‐specific fatty acid composition. We conducted a genome‐wide association study (GWAS) and fine mapping to identify genetic loci associated with fatty acid traits in each depot and assess their links to cardiometabolic diseases. We also explored whether these genetic associations were shared between SAT and VAT or were depot specific. A better understanding of these genetic factors could shed light on the biological mechanisms regulating lipid metabolism and inform future research on personalized dietary interventions and therapeutic strategies for cardiometabolic diseases.

## 2 Methods

### 2.1 MRI‐Derived Estimates of Fatty Acid Composition

This study utilized MRI‐derived estimates of fatty acid composition in SAT and VAT obtained from 33,583 participants in the UK Biobank. MRI data were obtained using a Siemens Aera 1.5‐T MRI scanner (Syngo MR D13; Siemens, Erlangen, Germany), as described in the UK Biobank abdominal MRI protocol [14]. MRI data acquisition included neck‐to‐knee chemical‐shift‐based imaging and pancreas single‐slice multiecho sequences with the following parameters: repetition time (TR): 27 ms; echo times (TEs): 2.38, 4.76, 7.15, 9.53, 11.91, 14.29, 16.67, 19.06, 21.44, and 23.82 ms; flip angle: 20°; bandwidth: 710 Hz; voxel size: 2.5 × 2.5 × 6.0 mm; matrix size: 160 × 160 [15]. SAT and VAT segmentations were generated using three‐dimensional chemical‐shift‐encoded MRI sequences processed with a deep learning algorithm [16]. These segmentations were projected onto the single‐slice multiecho images to extract depot‐specific metrics.

Fatty acid composition was quantified using a method adapted from Bydder et al. [17]. This approach characterizes triglyceride molecules based on the number of double bonds (NDB), methylene‐interrupted double bonds (NMIDB), and chain length. NDB was estimated using a nonlinear least‐squares fit of the MRI signal as a function of TEs, with NMIDB calculated using the relationship NMIDB = 0.093 × NDB^2^. Constraints for NDB were set at 1 ≤ NDB ≤ 6. Fatty acid composition fractions were derived as follows: unsaturated fatty acid fraction (fUFA) = (NDB − NMIDB)/3, saturated fatty acid fraction (fSFA) = 1 − fUFA, and monounsaturated and polyunsaturated fatty acid fraction (fMUFA + fPUFA) = 1 − fSFA. Voxel‐by‐voxel fatty acid composition maps were generated, and SAT and VAT segmentations were applied to isolate these compartments. Postprocessing steps included binary erosion of SAT and VAT masks to minimize partial‐volume effects and exclusion of voxels with fat fractions below 20% [17, 18] (online Supporting Information Methods and Figure S1).

### 2.2 Genetic Association Analysis

GWAS was performed separately for each of the six fatty acid traits in both SAT and VAT (fSFA, fMUFA, fPUFA) using REGENIE version v3.1.1 [19]. Our GWAS included participants self‐identified as “White British” who clustered with this group in principal component analysis. We excluded participants with sex chromosome anomalies, sex discrepancies, heterozygosity outliers, and genotype call rate outliers. Covariates included age, squared age, sex, genotyping array, imaging center, and the first 10 genotype‐related principal components. Each fatty acid trait was inverse normal transformed before analysis. Imputed SNPs, filtered by a minor allele frequency (MAF) > 0.01 and an INFO score > 0.9, resulted in 9,788,243 SNPs for the final analysis. The UK Biobank field codes used for sample and SNP quality control are provided in Table S1.

To determine whether the effects of the identified genetic loci differed between fat depots, we compared the genetic associations for matched fatty acid traits between SAT and VAT (e.g., SAT‐fSFA vs. VAT‐fSFA, SAT‐fMUFA vs. VAT‐fMUFA, and SAT‐fPUFA vs. VAT‐fPUFA). We assessed statistical heterogeneity using Cochran's Q test and calculated *I* ^2^ statistics (HetIsq). Loci with a heterogeneity *p* value (HetPval) < 0.05 and *I* ^2^ > 75% were considered to present depot‐specific effects.

### 2.3 Association With Disease Outcomes

We examined the effects of fatty acid‐associated genetic loci on 10 disease outcomes including type 2 diabetes, metabolic dysfunction‐associated steatotic liver disease (MASLD), hypertension, coronary artery disease, stroke, myocardial infarction, peripheral artery disease, deep vein thrombosis, pulmonary embolism, and cholithiasis. We obtained two sets of genome‐wide summary level data for the 10 outcomes from FinnGen data freeze 10 except for peripheral artery disease (data freeze 7) and other published GWAS analyses. We meta‐analyzed the effect of fatty acid‐associated variants on these 10 disease outcomes. Details of the disease definitions and sample sizes are summarized in Table S2.

### 2.4 Functional and Positional Analysis

To identify potentially causal variants within each associated genomic locus, we conducted fine mapping using a Bayesian framework implemented via *susieR*, a summary‐statistics‐based fine‐mapping R package. This approach allows for the estimation of the probability that each variant within a locus is causally linked to the observed trait association. Variants with a posterior inclusion probability (PIP) ≥ 95% were prioritized as likely causal candidates [20].

For colocalization analysis, we used coloc, a Bayesian statistical method designed to assess the probability that two traits share a causal variant using GWAS summary statistics [21]. To define the genomic region for analysis, we selected a 200‐kilobase (kb) window around the lead SNP, which was identified as the variant with the lowest *p* value in the GWAS. Colocalization analysis was conducted at genomic loci showing strong statistical evidence of association (*p* ≤ 5 × 10^−8^) with disease outcomes. To further refine the set of independent variants, we utilized PLINK 1.9 to remove variants in high linkage disequilibrium ([LD] threshold *r* ^2^ > 0.5), thereby reducing redundancy and avoiding dilution of shared causal signals [22]. We used coloc default priors to estimate the posterior probability of colocalization, considering PP.H4 (the probability that the two traits share a causal variant) as the primary measure of colocalization strength. Genetic variants with PP.H4 ≥ 0.95 were considered strong candidates for shared genetic effects between the two traits. To assess the robustness of our findings, sensitivity analysis was performed at a threshold of > 70%, ensuring that colocalization results remained consistent under different assumptions about the proportion of shared causal variants.

We utilized FUMA v1.5.2 with default MAGMA v1.5.2 settings [23]. We used the GTEx v8 project to evaluate variant effects on gene expression in relevant tissues (expression quantitative trait loci (eQTL)). To further enhance the biological interpretation of our findings, we incorporated protein quantitative trait loci (pQTL) data from the UK Biobank Pharma Proteomics Project (UKB‐PPP) [24]. Independent significant SNPs at the genomic risk loci for each SAT and VAT fatty acid were mapped to cis pQTL to provide additional evidence for the biological relevance of the identified genetic associations.

### 2.5 Gene–Diet Interaction Analysis

To explore whether genetic variation in adipose tissue fatty acid composition modifies the association between dietary fatty acid intake (SFA, MUFA and PUFA) and cardiometabolic disease risk (hypertension, cardiovascular disease, and type 2 diabetes), we performed a gene–environment interaction analysis using the lead variant at the most strongly associated locus for SAT and VAT fatty acids as identified by our GWAS and fine‐mapping analyses. Logistic regression models included main effects for genotype and dietary intake, as well as their interaction term (e.g., interaction term = genotype × dietary intake). The model was adjusted for age and sex.

Disease phenotypes and dietary intake variables were derived from UK Biobank data, incorporating both hospital episode statistics (HES) and self‐reported records. Participants were classified as cases if they had a recorded diagnosis (ICD‐10 codes) or a self‐reported history of the condition prior to their first imaging visit. Hypertension was defined based on one or more of the following: self‐reported diagnosis, use of antihypertensive medication, or elevated blood pressure (≥ 140/90 mmHg). Type 2 diabetes was identified using ICD‐10 codes and self‐reported diabetes diagnoses. Cardiovascular disease included diagnoses of angina, myocardial infarction, chronic ischaemic heart disease, atrial fibrillation, heart failure, or stroke, based on ICD‐10 codes. Medication use was captured from self‐reported treatment fields, with antihypertensive medication use defined by regular reporting of blood pressure medications. All relevant disease and medication codes used in phenotype definitions are detailed in Table S3.

### 2.6 Association With Plasma Fatty Acids Traits

To characterize the association between genetic loci linked to SAT and VAT fatty acid composition and circulating plasma fatty acid levels, we examined their effects on plasma SFAs, MUFAs, PUFAs, omega‐3 and omega‐6 fatty acids, linoleic acid, docosahexaenoic acid (DHA), degree of unsaturation, total fatty acids (total FAs), and several fatty acid ratios (e.g., MUFAs/total FAs, PUFAs/MUFAs, PUFAs/SFAs, omega‐6/total FAs). We used summary‐level data from the Nightingale metabolites GWAS, which includes 249 metabolite measures in up to 114,000 individuals of European ancestry from the UK Biobank [25]. All associations were aligned to the SAT fSFA‐decreasing allele, and statistical significance was determined using a false discovery rate (FDR) threshold adjusted for 17 tests (*p* < 0.003).

## 3 Results

We analyzed MRI data from 33,583 UK Biobank participants to estimate fSFA, fMUFA, and fPUFA in SAT and VAT. These values represent model‐based estimates derived from MRI signal fitting and not direct measurements of individual fatty acids. The baseline characteristics of the participants, including age (Field 21003), sex (Field 31), body mass index (BMI) (Field 21001), weight (Field 21002), height (Field 50), SFAs (Field 26014), MUFAs (Field 26032), PUFAs (Field 100007), and other demographic variables, are summarized in Table 1. In both men and women, VAT had significantly higher fSFA and fPUFA but lower fMUFA compared to SAT (*p* < 0.0004; Table 1). When comparing sexes, women had higher fMUFA and fPUFA and lower fSFA in SAT than men (*p* < 0.05). In contrast, VAT in women showed lower fMUFA and fPUFA and higher fSFA than in men (*p* < 0.05).

**TABLE 1:** Baseline characteristics of UK Biobank participants included in the analysis, stratified by sex.

	Combined	Women	Men
Number	33,583	17,264	16,319
Age (years)	64.47 ± 7.68	63.80 ± 7.50	65.17 ± 7.80**
BMI (kg/m^2^)	26.46 ± 4.25	26.04 ± 4.64	26.89 ± 3.74**
Dietary MUFA (g)	26.96 ± 10.73	25.08 ± 9.65	28.96 ± 11.44**
Dietary PUFA (g)	19.90 ± 7.51	18.77 ± 6.92	21.11 ± 7.92**
Dietary SFA (g)	27.54 ± 11.78	25.57 ± 10.67	29.64 ± 12.52**
Total energy (kJ)	8764.34 ± 2372.48	8099.30 ± 2052.21	9475.21 ± 2483.10**
Total fat (g)	74.40 ± 27.93	69.43 ± 25.18	79.71 ± 29.69**
Hypertension	12,093 (36%)	4946 (28.6%)	7147 (43.8)**
Type 2 diabetes	1765 (5.3%)	586 (3.4%)	1179 (7.3%)**
Cardiovascular disease	3306 (9.8%)	977 (5.7%)	2329 (14.3%)**
Subcutaneous adipose tissue (SAT) fatty acid composition			
fSFA	0.44 ± 0.04	0.43 ± 0.03	0.45 ± 0.04**
fMUFA	0.40 ± 0.03	0.41 ± 0.02	0.40 ± 0.03**
fPUFA	0.15 ± 0.05	0.16 ± 0.04	0.15 ± 0.05**
Visceral adipose tissue (VAT) fatty acid composition			
fSFA	0.47 ± 0.04	0.48 ± 0.04	0.46 ± 0.03**
fMUFA	0.36 ± 0.03	0.35 ± 0.03	0.37 ± 0.03**
fPUFA	0.18 ± 0.04	0.17 ± 0.04	0.18 ± 0.03**

Abbreviations: MUFA, monounsaturated fatty acids; PUFA, polyunsaturated fatty acids; SFA, saturated fatty acids.

** Statistical significance after Bonferroni correction (threshold *p* < 0.004), accounting for multiple comparisons.

### 3.1 Genetic Associations With SAT and VAT Fatty Acid Composition

**FIGURE 1:**
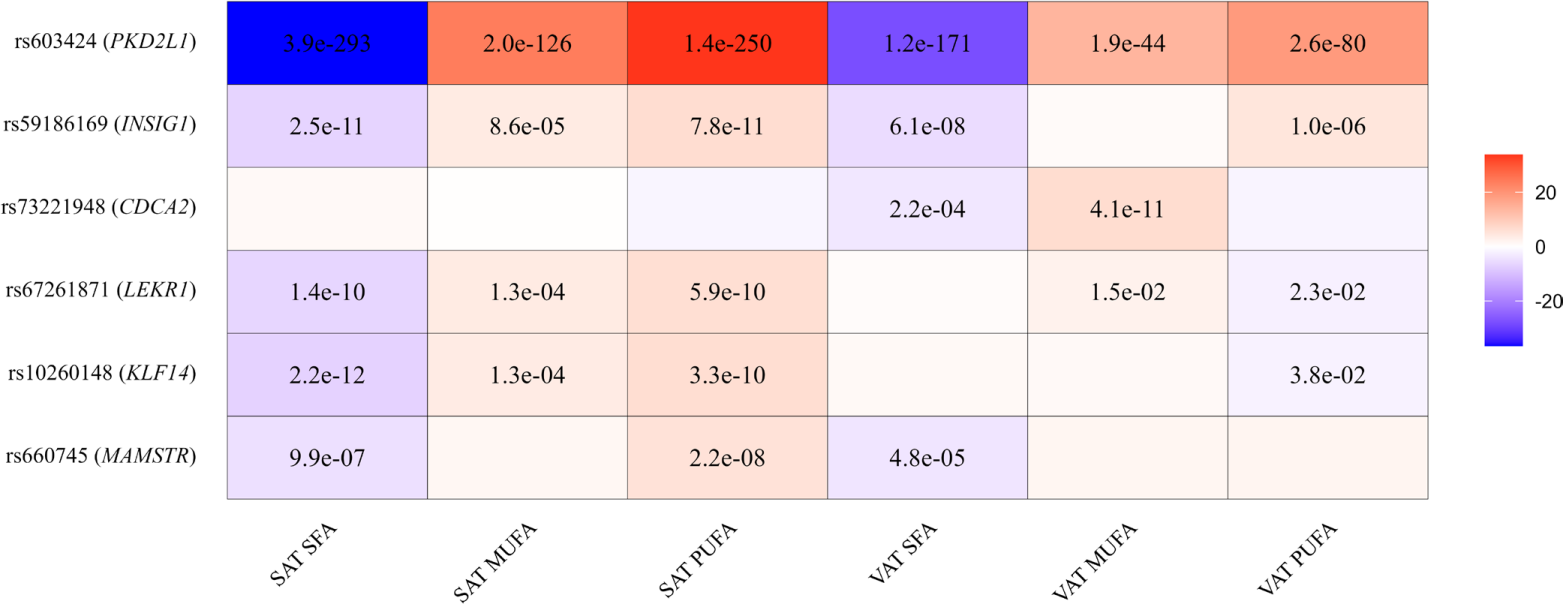
Associations between six genetic loci and fatty acid composition in subcutaneous (SAT) and visceral adipose tissue (VAT). Effects are shown for saturated (fSFA), monounsaturated (fMUFA), and polyunsaturated fatty acids (fPUFA). The color and intensity of each cell indicate the direction and magnitude of the effect, as estimated by linear regression in the genome‐wide association model. Numbers within cells represent the corresponding *p* values. All effects are aligned to the allele associated with decreased fSFA in SAT or VAT. [Color figure can be viewed at wileyonlinelibrary.com]

We identified six genetic loci associated with one or more SAT and VAT fatty acid traits (Figure 1, Table 2, and Figure S2). These associations include rs603424‐G (near *PKD2L1*) associated with higher fMUFA, fPUFA, and lower fSFA in both SAT and VAT, rs59186169‐A (*INSIG1*) associated with higher fPUFA and lower fSFA in both SAT and VAT, rs73221948‐G (*CDCA2*) associated with higher fMUFA in VAT, rs67261871‐T (*LEKR1*) associated with higher fMUFA and fPUFA and lower fSFA in SAT, rs10260148‐C (*KLF14*) associated with higher fPUFA and lower fSFA in SAT, and rs660745‐T (*MAMSTR*) associated with higher fPUFA in SAT.

**TABLE 2:** Effects of six genetic loci associated with subcutaneous (SAT) and visceral adipose tissue (VAT) fatty acid composition.

rsID	CHR	BP	EA	OA	BETA	SE	*p*	EAF	N	HetPval	Phenotype	Nearest gene
rs10260148	7	130,430,969	C	T	−0.061	0.009	2.E‐12	0.72	28,402	7.E‐04	SAT fSFA	*KLF14*
rs59186169	7	155,050,209	A	G	−0.116	0.017	3.E‐11	0.95	28,402	4.E‐01	SAT fSFA	*INSIG1*
rs603424	10	102,075,479	G	A	−0.386	0.011	4.E‐293	0.83	28,402	9.E‐10	SAT fSFA	*PKD2L1*
rs660745	19	49,219,459	T	C	0.044	0.007	2.E‐08	0.46	28,402	1.E‐02	SAT fPUFA	*MAMSTR*
rs67261871	3	156,797,941	T	C	−0.051	0.008	1.E‐10	0.6	28,402	5.E‐05	SAT fSFA	*LEKR1*
rs73221948	8	25,464,670	G	T	0.06	0.009	4.E‐11	0.71	28,413	6.E‐06	VAT fMUFA	*CDCA2*

Abrreviations: BP, position (build 37); CHR, chromosome; EA, effect allele; N, sample size; OA, other allele; rsID, SNP identifier; SE, standard error; Phenotype, fatty acid trait showing the strongest association at each locus; saturated (fSFA), monounsaturated (fMUFA), and polyunsaturated (fPUFA) fatty acids.

To assess depot‐specific effects, we conducted heterogeneity analysis comparing genetic associations across SAT and VAT for each fatty acid trait. Significant heterogeneity indicated depot‐specific associations for several loci: rs67261871 (HetPval = 5.1 × 10^−5^) and rs10260148 (HetPval = 4.6 × 10^−5^) showed strong evidence of SAT‐specific effects on fSFA; rs73221948 (HetPval = 7.1 × 10^−4^) had VAT‐specific effects on fMUFA, and rs660745 (HetPval = 0.01) had SAT‐specific effects on fPUFA (Table 2).

### 3.2 Associations Between Fatty Acids Genetic Loci and Disease Outcomes

**FIGURE 2:**
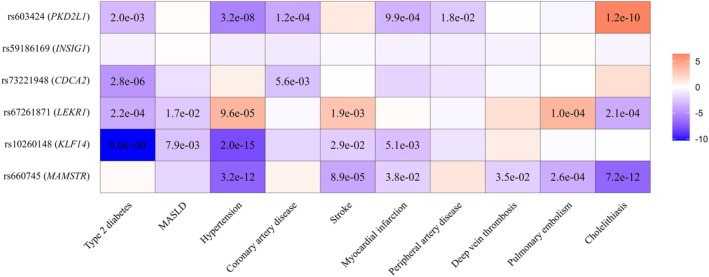
Effect of six genetic loci associated with subcutaneous (SAT) and visceral adipose tissue (VAT) fatty acid composition on 10 disease outcomes. Each cell represents the direction and magnitude of the genetic association (based on meta‐analyses of FinnGen and publicly available GWAS data). Colors reflect effect size and direction, while numbers within cells indicate the corresponding *p* values from regression analyses. All effects are aligned to the allele associated with lower saturated fatty acids in SAT or VAT. [Color figure can be viewed at wileyonlinelibrary.com]

We evaluated the associations between the fSFA‐lowering alleles of the six lead genetic loci and ten cardiometabolic disease outcomes. Several loci demonstrated significant associations with disease risk (Figure 2 and Table S4): rs603424‐G (*PKD2L1*) was associated with lower risk of hypertension (*p* = 3.2 × 10^−8^) and coronary artery disease (1.2 × 10^−4^) and higher risk of cholithiasis (1.2 × 10^−10^); rs73221948‐G (*CDCA2*) was associated with lower risk of type 2 diabetes (2.8 × 10^−6^); rs67261871‐T (*LEKR1*) was associated with lower risk of type 2 diabetes (2.2 × 10^−4^) and cholelithiasis (2.1 × 10^−4^) and higher risk of hypertension (9.6 × 10^−5^) and pulmonary embolism (1 × 10^−4^); rs10260148‐C (*KLF14*) was associated with lower risk of type 2 diabetes (*p* < 10^−150^) and hypertension (*p* = 2 × 10^−15^); and rs660745‐T (*MAMSTR*) was associated with lower risk of hypertension (*p* = 3.2 × 10^−12^), stroke (*p* = 8.9 × 10^−5^), pulmonary embolism (*p* = 2.6 × 10^−4^), and cholithiasis (*p* = 7.2 × 10^−12^).

### 3.3 Fine Mapping and Colocalization Analysis

To identify potential causal variants underlying the GWAS signals, we performed fine‐mapping analyses. We prioritized variants with a high PIP ≥ 0.95. This approach identified rs603424 on chromosome 10 (Figure S3) as the most likely causal variant, with a single‐variant credible set and the strongest association with fSFA in both SAT and VAT (Table S5). Functional annotation provided further insight into the biological relevance of this variant. The rs603424‐G allele, associated with lower fSFA, was linked to higher expression of *SCD1* in both VAT (multiple test correct *p* value = 2 × 10^−6^) and SAT (2 × 10^−6^) and lower expression of *PKD2L1* in the brain frontal cortex (1.3 × 10^−6^) (Table S6).

We performed colocalization analyses at genomic regions showing significant disease associations. At the *PKD2L1* locus, rs603424 showed strong evidence of a shared genetic signal with both hypertension and cholelithiasis, suggesting that this variant may drive both fatty acid composition and disease risk at this locus. At the *KLF14* locus on chromosome 7, colocalization analyses indicated that the same genetic region likely influences both type 2 diabetes and hypertension. Within this region, rs12154627 emerged as the most likely shared causal variant for both traits. At the *MAMSTR* locus on chromosome 19, we also observed a shared genetic signal between the fatty acid trait and hypertension, with rs479486 identified as the most probable causal variant (Table S7 and Figures S4–S9).

### 3.4 Gene–Diet Interaction Analysis

**FIGURE 3:**
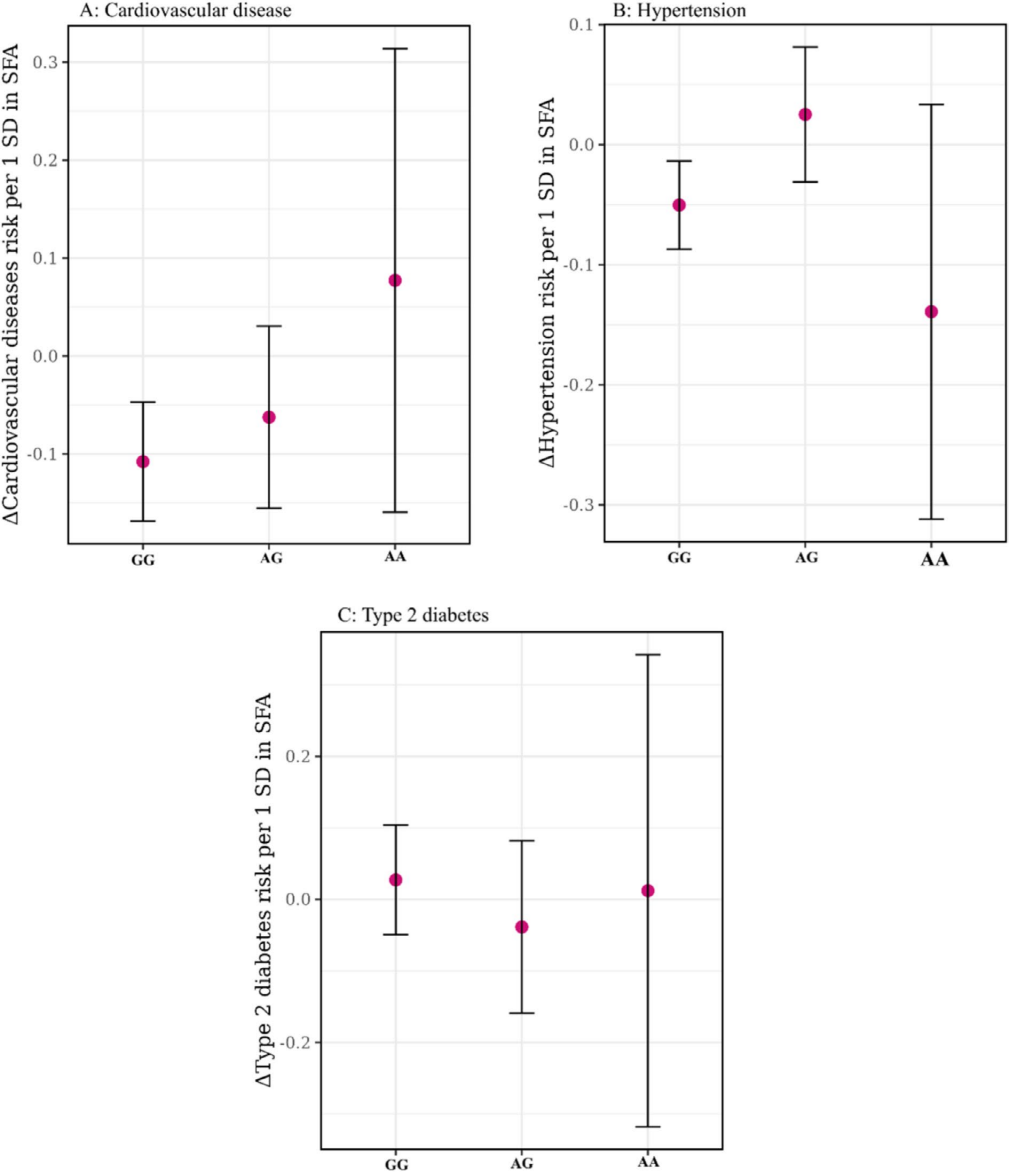
Interaction between rs603424 genotype and dietary saturated fatty acid (SFA) intake on cardiometabolic disease risk. Effect estimates represent the change in disease risk (log odds ratio) per 1 standard deviation increase in dietary SFA intake for (A) cardiovascular disease, (B) hypertension, and (C) type 2 diabetes, stratified by rs603424 genotype (GG, AG, AA). Associations were estimated using logistic regression models adjusted for age, sex, and ethnic background. Points represent genotype‐specific effect sizes, and error bars indicate 95% confidence intervals. [Color figure can be viewed at wileyonlinelibrary.com]

We tested whether the genetic effect of rs603424 modifies the relationship between dietary fatty acid intake and cardiometabolic disease risk. Interaction terms between rs603424 and standardized intake of SFAs, MUFAs, and PUFAs were included in logistic regression models for hypertension, type 2 diabetes, and cardiovascular disease. Overall, no statistically significant gene–diet interactions were observed for most outcomes (*p* > 0.05). However, a nominally significant interaction was detected between rs603424 and dietary SFA intake on cardiovascular disease (*β* = 0.0073, SE = 0.0036, *p* = 0.041), suggesting a potential modifying effect of this genotype on the relationship between SFA intake and cardiovascular disease risk. This finding warrants cautious interpretation given the marginal *p* value. No meaningful interactions were observed for type 2 diabetes or hypertension across any of the fatty acid categories (all *p* > 0.18) (Figure 3 and Table S8).

### 3.5 Associations With Circulating Fatty Acids

**FIGURE 4:**
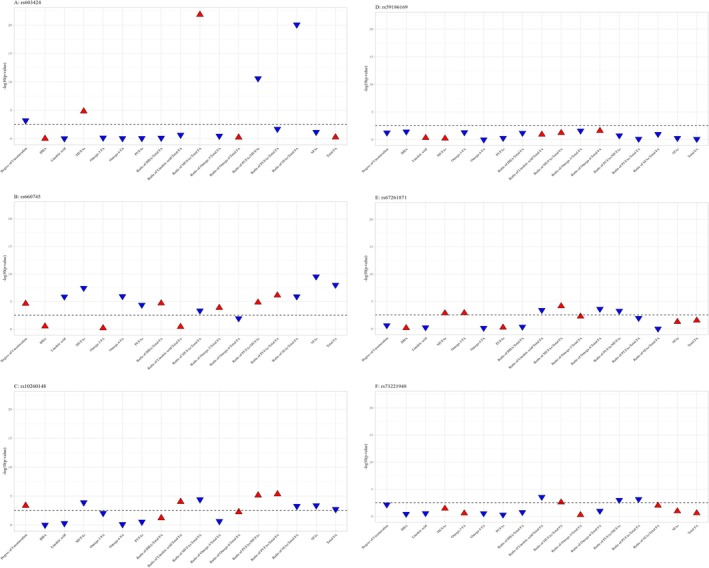
Associations between six genetic loci linked to adipose tissue fatty acid composition and 17 circulating plasma fatty acid traits. The x‐axis represents 17 plasma fatty acid traits derived from the Nightingale metabolomics GWAS, and the y‐axis shows the –log_10_(*p* value) from linear regression analyses. The horizontal dashed line denotes the significance threshold after false discovery rate (FDR) correction (*p* < 0.003). Each data point represents the association of a single SNP with a given plasma fatty acid trait, with red and blue triangles indicating the direction of effect: red for a positive association and blue for a negative association. All associations are aligned to the subcutaneous or visceral adipose tissue saturated fatty acid‐decreasing allele of each variant. [Color figure can be viewed at wileyonlinelibrary.com]

We examined the associations between the six genetic loci linked to adipose tissue fatty acid composition and 17 plasma fatty acid traits (Figure 4). The fSFA‐decreasing allele of rs603424 (*PKD2L1*) was associated with higher plasma levels of MUFAs and the MUFAs/total FAs ratio and lower values for degree of unsaturation, PUFAs/MUFAs, and SFAs/total FAs ratios. In addition, rs660745 (*MAMSTR*) showed broad associations, including increased degree of unsaturation, higher levels of plasma PUFAs and the ratios of DHA/total FAs, omega‐3/total FAs, PUFAs/MUFAs, and PUFAs/total FAs, and lower plasma levels of linoleic acid, omega‐6, SFAs, total fatty acids, and several ratios including MUFAs/total FAs, omega‐6/total FAs, and SFAs/total FAs.

## 4 Discussion

Our study presents the first large‐scale GWAS of fatty acid composition in human adipose tissue depots, using noninvasive MRI‐derived estimates in 33,583 UK Biobank participants. We identified six genetic loci associated with fatty acid fractions in SAT and VAT, revealing shared and depot‐specific regulation. These findings offer new insights into the genetic architecture of adipose lipid composition and its implications for cardiometabolic disease risk.

The most compelling signal emerged at the *PKD2L1* locus, which was associated with fSFA, fMUFA, and fPUFA across both SAT and VAT. Fine‐mapping prioritized rs603424 as the likely causal variant, and colocalization analyses linked it to hypertension and cholelithiasis. Functional annotation revealed rs603424 to be an eQTL for *SCD1* in adipose tissue—an enzyme catalyzing the conversion of SFAs to MUFAs [26]—suggesting a direct mechanistic link between the genetic signal and lipid desaturation. This aligns with observed cardiometabolic protection [27]: The fSFA‐decreasing allele was associated with lower risk of type 2 diabetes, coronary artery disease, myocardial infarction, and hypertension. Moreover, the effect of rs603424‐G on plasma fatty acid levels supports the role of *SCD1* in mediating fatty acid remodeling, as evidenced by the increase in circulating MUFAs and a decrease in SFAs. Overexpression of *SCD1* in adipose tissue promotes fat mobilization and energy expenditure [28]. The increased risk of cholelithiasis associated with rs603424‐G highlights a potential biological trade‐off. Although SCD1‐mediated desaturation protects against cardiometabolic diseases, it may simultaneously promote gallstone formation potentially via changes in bile lipid composition and cholesterol solubility [29].

Gene–diet interaction analysis suggested that individuals carrying the rs603424‐A allele—associated with higher fSFA—may experience greater cardiovascular risk in the context of high dietary SFA intake. This supports the hypothesis that SCD1‐mediated desaturation moderates the deleterious effects of saturated fats, with protective effects most evident in genetically predisposed individuals presenting higher enzymatic activity [30].

Other loci identified presented distinct and biologically plausible effects. The *INSIG1* variant (rs59186169‐A) was associated with lower fSFA and higher fPUFA in both depots, consistent with INSIG1's role in regulating SREBP and downstream fatty acid desaturation [31]. Despite favorable lipid profiles, this variant showed no associations with cardiometabolic disease, in line with previous murine studies suggesting minimal systemic metabolic impact [32].

Variants at *KLF14* (rs10260148‐C) and *MAMSTR* (rs660745‐T) were both associated with more favorable lipid compositions in SAT and linked to reduced risk of multiple cardiometabolic outcomes. KLF14, a known master regulator of adipose biology and insulin sensitivity [33], has previously been implicated in sex‐specific metabolic effects [34]. Colocalization analysis at this locus pointed to rs12154627 as the most likely causal variant.

The circulating fatty acid profiles linked to these loci help to reconcile some of the paradoxical findings. For instance, although the fSFA‐decreasing alleles of rs603424 and rs67261871 (*LEKR1*) were associated with cardiometabolic protection, they also increased circulating MUFAs and reduced the degree of unsaturation—profiles that have been implicated in hepatic steatosis [35] and gallstone formation [29]. Conversely, loci like *KLF14* and *MAMSTR* were associated with higher plasma PUFA levels and greater unsaturation, consistent with anti‐inflammatory and lipid‐lowering effects [36].

These divergent patterns underscore the complexity of lipid metabolism and its interplay between adipose tissue, liver, and systemic circulation. Our results suggest that favorable fatty acid profiles within adipose tissue—particularly reduced SFA content—may not always translate into equally beneficial systemic effects, depending on how lipids are mobilized and redistributed.

Fatty acid composition in adipose tissue is influenced by a complex interplay between dietary intake, systemic lipid metabolism, and depot‐specific storage mechanisms. While our study focuses on the genetic determinants of fatty acid composition in SAT and VAT, previous work has shown that habitual dietary intake of saturated and unsaturated fats is reflected in these depots, with significant correlations to circulating lipid biomarkers and metabolic disease risk [18]. Our findings suggest that depot‐specific fatty acid profiles are not only heritable but may also mediate cardiometabolic outcomes through pathways involving lipid desaturation and storage efficiency. Future studies integrating dietary, circulating, and tissue‐level lipidomic data will be valuable for understanding how these pathways interact and how they might be modifiable to reduce disease risk.

A major strength of this study is the integration of imaging, genomics, and functional data, which provides a high‐resolution, noninvasive approach to dissect depot‐specific lipid metabolism in humans. This approach enables novel insight beyond conventional plasma‐based lipid phenotyping, and our identification of depot‐specific signals (e.g., VAT‐specific effects at *CDCA2*) reflects the metabolic heterogeneity of adipose depots.

Several limitations must be acknowledged. First, our findings are based on indirect estimates of fatty acid composition derived from MRI modeling parameters (NDB and NMIDB), rather than direct quantification from tissue biopsies. While this approach allows for large‐scale, noninvasive phenotyping, it does not provide absolute concentrations of specific fatty acids. Therefore, our interpretation focuses on relative differences and genetic influences across depots. Second, we were unable to conduct MR analysis due to the limited number of instrumental variables available for fatty acid traits. Although GWAS analyses offer valuable insights, they do not establish causality, and future studies should aim to develop stronger genetic instruments to enable MR‐based causal inference. Third, our findings are based predominantly on White British individuals, which may limit their generalizability to other ethnic groups. Given that genetic variation, dietary habits, and environmental factors differ across populations, expanding this research to more diverse cohorts will be crucial for validating the replicability and transferability of these associations. Fourth, while MRI‐based fatty acid estimates provide high spatial resolution, they are still subject to technical variability and potential confounding factors, such as MRI signal noise, partial volume effects, and individual hydration status or fat composition differences, which could influence the accuracy of fatty acid fraction estimates. Future studies should incorporate independent validation datasets to improve the precision of fatty acid quantification in adipose tissue. Lastly, although our GWAS, fine mapping, and colocalization analyses suggest that *SCD1* plays key regulatory roles in fatty acid metabolism, their biological functions remain incompletely understood. Functional studies will be essential to confirm causal effects and determine how these loci influence fatty acid synthesis, storage, and metabolism in adipose tissue.

## 5 Conclusion

We demonstrated that common genetic variation contributes to individual differences in adipose tissue fatty acid composition, with implications for cardiometabolic health. Our findings highlight the potential for genetically informed dietary interventions targeting lipid metabolism, and open new avenues for personalized nutrition and disease prevention based on adipose lipid biology.

## Author Contributions

A.A. and A.N. analyzed the data. H.Y. designed the study. A.A. and H.Y. wrote the manuscript. M.C., B.W., M.T., E.P.S., E.L.T., and J.D.B. provided all the MRI derived. M.C. performed the GWAS. All authors contributed to reviewing, editing, and approving the manuscript. H.Y. is the guarantor of this work and, as such, has full access to all the data in the study and takes responsibility for the integrity of the data and the accuracy of the data analysis.

## Conflicts of Interest

M.C. and E.P.S. are employees of Calico Life Sciences LLC. The other authors declare no conflicts of interest.

## Supporting information


**Data S1:** oby70045‐sup‐0001‐supinfo.docx.

## Data Availability

Our research was conducted using UK Biobank data. Under the standard UK Biobank data sharing agreement, we (and other researchers) cannot directly share raw data obtained or derived from the UK Biobank. However, under this agreement, all of the data generated and methodologies used in this paper are returned by us to the UK Biobank, where they will be fully available. Access can be obtained directly from the UK Biobank to all bona fide researchers upon submitting a health‐related research proposal to the UK Biobank https://www.ukbiobank.ac.uk.
