# Supplementary material for: Genetic Determinants of Fatty Acid Composition in Subcutaneous and Visceral Adipose Tissue
Source: Obesity (Silver Spring). 2025 Sep 29;33(12):2406–15. doi: 10.1002/oby.70045 (PMC12636063; doi:10.1002/oby.70045)
Supplement: Supplementary file 1 — Data S1: oby70045‐sup‐0001‐supinfo.docx. [file OBY-33-2406-s001.docx]

**Genetic determinants of fatty acid composition in subcutaneous and visceral adipose tissue**

Altayeb Ahmed, School of Natural Sciences, College of Health and Science, University of Lincoln, Joseph Banks Laboratories, Green Lane, Lincoln, UK., Email: [28404091@students.lincoln.ac.uk](mailto:28404091@students.lincoln.ac.uk)

Afreen Naz, School of Natural Sciences, College of Health and Science, University of Lincoln, Joseph Banks Laboratories, Green Lane, Lincoln, UK., Email: [ANaz@lincoln.ac.uk](mailto:ANaz@lincoln.ac.uk)

Marjola Thanaj, Research Centre for Optimal Health, School of Life Sciences, University of Westminster, London, UK., Email: [m.thanaj@westminster.ac.uk](mailto:m.thanaj@westminster.ac.uk)

Elena P Sorokin, Calico Life Sciences LLC, South San Francisco, CA, United States., Email: [sorokin@calicolabs.com](mailto:sorokin@calicolabs.com)

Brandon Whitcher, Research Centre for Optimal Health, School of Life Sciences, University of Westminster, London, UK., Email: [b.whitcher@westminster.ac.uk](mailto:b.whitcher@westminster.ac.uk)

Jimmy D Bell, Research Centre for Optimal Health, School of Life Sciences, University of Westminster, London, UK., Email: [J.Bell@westminster.ac.uk](mailto:J.Bell@westminster.ac.uk)

E Louise Thomas, Research Centre for Optimal Health, School of Life Sciences, University of Westminster, London, UK., Email: [l.thomas3@westminster.ac.uk](mailto:l.thomas3@westminster.ac.uk)

Madeleine Cule, Calico Life Sciences LLC, South San Francisco, CA, United States., Email: [cule@calicolabs.com](mailto:cule@calicolabs.com)

Hanieh Yaghootkar^1,2^

^1^School of Natural Sciences, College of Health and Science, University of Lincoln, Joseph Banks Laboratories, Green Lane, Lincoln, UK., Email: [HYaghootkar@lincoln.ac.uk](mailto:HYaghootkar@lincoln.ac.uk)

^2^Human Development and Health, Faculty of Medicine, University of Southampton, Southampton, United Kingdom., Email: [h.yaghootkar@soton.ac.uk](mailto:h.yaghootkar@soton.ac.uk).

**Corresponding author:**

Hanieh Yaghootkar, Human Development and Health, Faculty of Medicine, University of Southampton, Southampton, United Kingdom., Email: h.yaghootkar@soton.ac.uk

**Supplementary Method**

**Data Analysis**

The MRI-based method used to quantify adipose tissue FA composition in this study is based on nonlinear least-squares curve-fitting procedure to estimate the number of double bonds (NDB) in triglyceride molecules from multi-echo MRI data, as previously described by on Bydder et al. (1). The model captures triglyceride composition by fitting both real and imaginary components of the signal using phase-corrected multiecho chemical-shift-encoded MRI.

The MRI signal S(t) was fitted voxel-wise using a complex model:

S(t) = [w · a(t) + f · b(t)] · exp(iϕ) · exp(−R2*t) · exp(iΔB0·t)

a(t) = awater · exp(i·ω·δwater·t)

b(t) = Σ aj(ndb) · exp(i·δj·t)

Where:

- awater and aj represent the number of protons in water and fat.
- δj represents the chemical shifts,
- ω = 2π · 42.576×10⁶ · B0 (rad/s) is the Larmor frequency.

- Parameters were field inhomogeneity (ΔB0), transverse decay (R2⁎), initial phase (ϕ), ndb, water (w) and fat (f), and all were real numbers. The MR signal was modelled as a complex-valued function of echo time (TE), accounting for signal magnitude, B0 inhomogeneity, and phase evolution. Among them, ΔB0, R2*, and ndb were determined iteratively, while the remaining parameters were calculated algebraically. Supplementary Figure S1 shows an example of the maps of magnitude, field inhomogeneity (B0) and phase (φ) of the vegan and omnivore participants as well as the fitted complex-valued data for four representative voxels in the omnivore participant.

Model fitting was constrained to:

-1 ≤ NDB ≤ 6
 - NMIDB = 0.093 × NDB²

From these, the fatty acid fractions were derived as:

fMUFA = (NDB - 2 × NMIDB) / 3

fPUFA = NMIDB / 3

fUFA = (NDB - NMIDB) / 3

fSFA = 1 - fUFA = 1 - (fPUFA + fMUFA)

**Quality Control**

We applied a multi-step quality control pipeline to ensure robustness and reproducibility of fatty acid composition estimates (2). Voxel-wise fitting was applied within VAT and ASAT masks. Bound constraints were imposed to ensure physiologically meaningful NDB values (1 ≤ NDB ≤ 6). After post-processing SAT and VAT masks, we excluded 197 participants with no usable voxels remaining in either fat depot. Visual inspection confirmed signal dropout or segmentation failure. An additional 43 participants were excluded after inspecting the tails of the NDB distribution (outside acceptable range: 1 < NDB < 6). For consistency in the sample size, participants with missing data for one fat depot (e.g. ASAT) were excluded from the study even if the other fat depot (e.g. VAT) had full coverage.

**Supplementary table S1. UKBB fields used to perform the genome wide association study for sample/SNP quality control**

| **QC Step** | **UK Biobank Field Description** | **Field Code** | **Filter** |
| --- | --- | --- | --- |
| Genetic sex | Genetic sex | 22001 | Exclude if mismatch with reported sex |
| Reported sex | Sex | 31 | Compare with genetic sex |
| Sex chromosome aneuploidy | Sex chromosome aneuploidy | 22019 | Exclude non-XX or XY (e.g., XXY, X0) |
| Heterozygosity outliers | Heterozygosity | 22027 | Exclude ± SD from mean |
| Genotyping process and sample QC | Missing rate | 22005 | Exclude if missingness > 5% |
| Ancestry (in white British subset) | Genetic ethnic grouping | 22006 | Must be Yes |
| Genotype principal components | Genetic principal components 1–10 | 22009 | PC1–PC10 used as covariates |
| Genotyping array | Genotype measurement batch | 22000 | Include as a categorical covariate |
| Imaging center | Imaging centre | 54 | Include as covariate |
| Age | Age when attended imaging | 21003 | Include as covariate |
| Sex | Sex | 31 | Covariate |
| Imputation quality | INFO score from imputed dataset | — | Filter SNPs with INFO > 0.9 |
| Minor allele frequency (MAF) | Calculated from imputed data | — | Filter SNPs with MAF > 0.01 |

**Supplementary table. S2. Summary of GWAS datasets used for disease outcomes.** Disease outcome data was obtained from FinnGen and published GWAS in European population including both males and females. Phenocode: phenotype identifier; N Case: number of cases, N Control: number of controls, author: the author of the study for published GWAS (pGWAS) and year of publication; ICD 10 code: International disease definition code from FinnGen data freeze 10 and 7 for peripheral artery disease.

| **Trait/disease** | **Phenocode**  **Pubgwas** | **Phenocode**  **FinnGen** | **N Cases; Control (pGWAS)** | **N Case; Control (FinnGen)** | **Author (year)** | **PMID** | **ICD 10 code** |
| --- | --- | --- | --- | --- | --- | --- | --- |
| Type 2 diabetes | NA | T2D | 80154; 853816 | 65085;335112 | Mahajan et al. (2022) | 35551307 | ICD-10- E11 |
| MASLD | ebi-a-GCST90091033 | NAFLD | 8434;770180 | 2568;409613 | Ghodsian et al. (2021) | 34841290 | ICD-10- K76.0 |
| Hypertension | ebi-a-GCST90038604 | I9_HYPTENS | 129909;354689 | 122996;289117 | Dönertaş et al. (2021) | 33959723 | ICD-10- I10-I15, I67.4 |
| Coronary heart disease | ebi-a-GCST003116 | I9_ATHSCLE | 22233;64762 | 16243;381977 | Nickpay et al. (2015) | 26343387 | ICD-10- I70 |
| Stroke | ebi-a-GCST006908 | I9_STR | 34217;406111 | 27497;371723 | Malik et al. (2018) | 26343387 | ICD-10- I61, I63, I64 |
| Myocardial infarction | ebi-a-GCST011364 | I9_MI_STRICT | 14825;2680 | 26060;343079 | Hartiala et al. (2021) | 33532862 | ICD-10-I21, I22 |
| Peripheral artery disease | ebi-a-GCST90018890 | I9_PAD | 7114;475964 | 11924;288638 | Sakaue et al. (2021) | 34594039 | ICD-10- E10.5; E10.5+I79.2;E11.5, E11.5+I79.2; E12.5, E13.5; E14.5, I70.2, I73.9 |
| Deep vein thrombosis | ebi-a-GCST90038615 | I9_PHLETHROMBDVTLOW | 9529;475069 | 6501;357111 | Dönertaş et al. (2021) | 33959723 | ICD-10- I80.1, I80.20, I80.29 |
| Pulmonary embolism | ebi-a-GCST90013937 | I9_PULMEMB | 407;746 | 10046;401128 | Mbatchou et al. (2021) | 34017140 | ICD-10- I26 |
| Cholelithiasis | ebi-a-GCST90018819 | K11_CHOLELITH | 26122;461431 | 40191;361641 | Sakaue et al. (2021) | 34594039 | ICD-10-K80 |

**Supplementary table. S3. Summary of codes used to define dietary exposures and disease outcomes in the UK Biobank.** This table lists the specific field codes, phenotype definitions, and classification systems (e.g., ICD-10, UK Biobank field IDs) used to derive dietary intake variables and disease phenotypes in the study.

| **Trait** | **ICD-10** | **Self-Reported Code (20002)** | **UK Biobank Field** |
| --- | --- | --- | --- |
| Diet | - | - | 20086, 1389, 1369, 1359, 1349, 1379, 6144, 1418, 1408, 1428, 1329, 1339 |
| Hypertension | I10-I13, I15, O10 | 1065, 1072 | 6153, 6177 |
| Type 2 diabetes | E11 | 1220, 1223 | - |
| Cardiovascular disease | I20, I21, I25, I48, I50, I60, I61, I63, I64 | - | - |

**Supplementary table. S4. Associations between six genetic loci linked to subcutaneous (SAT) and visceral (VAT) adipose tissue fatty acid composition and ten cardiometabolic disease outcomes.** For each SNP–outcome pair, the table reports the meta-analysed effect size (Beta), standard error (SE), and p-value from genome-wide association analyses.

| **Outcome** | **rs660745-T** | **rs10260148-C** | **rs67261871-T** | **rs73221948-G** | **rs59186169-A** | **rs603424-G** |
| --- | --- | --- | --- | --- | --- | --- |
| Type 2 diabetes | 0.001; 0.005; 8E-1 | -0.052; 0.005; 0.0E+0 | -0.018; 0.005; 2E-4 | -0.025; 0.005; 3E-6 | -0.006; 0.011; 6E-1 | -0.02; 0.007; 2E-3 |
| MASLD | -0.026; 0.014; 7E-02 | -0.041; 0.016; 8E-3 | -0.035; 0.015; 2E-2 | -0.023; 0.016; 2E-1 | 0.005; 0.033; 9E-1 | 0.004; 0.02; 9E-1 |
| Hypertension | -0.006; 0.001; 3E-12 | -0.007; 0.001; 2E-15 | 0.003; 0.001; 1E-4 | 0.001; 0.001; 4E-1 | -0.001; 0.002; 4E-1 | -0.006; 0.001; 3E-8 |
| Coronary artery disease | 0.005; 0.008; 5E-1 | -0.015; 0.008; 7E-2 | -0.002; 0.008; 8E-1 | -0.029; 0.011; 6E-3 | -0.017; 0.017; 3E-1 | -0.037; 0.01; 1E-4 |
| Stroke | -0.032; 0.008; 9E-5 | -0.02; 0.009; 3E-2 | 0.026; 0.008; 2E-3 | 0.00; 0.011; 1E+00 | -0.006; 0.021; 8E-1 | 0.014; 0.012; 2E-1 |
| Myocardial infarction | -0.016; 0.007; 4E-2 | -0.023; 0.008; 5E-3 | 0.002;0.008; 8E-1- | -0.016; 0.009; 6E-2 | -0.011; 0.016; 5E-1 | -0.035; 0.011; 1E-3 |
| Peripheral artery disease | 0.015; 0.011; 2E-1 | -0.013; 0.011; 2E-2 | 0.004; 0.01; 7E-1 | -0.014; 0.012; 2E-1 | -0.02; 0.024; 4E-1 | -0.038; 0.016; 2E-2 |
| Deep vein thrombosis | -0.001; 0.0; 4E-2 | 0.00; 0.00; 3E-1 | 0.00; 0.00; 1E-1 | 0.00; 0.00; 8E-1 | 0.00; 0.001; 5E-1 | 0.00; 0.00; 9E-1 |
| Pulmonary embolism | -0.045; 0.012; 3E-4 | -0.002; 0.014; 9E-1 | 0.051; 0.013; 1E-4 | 0.00; 0.014; 1E+00 | 0.005; 0.028; 9E-1 | -0.008; 0.018; 7E-1 |
| Cholelithiasis | -0.041; 0.006; 7E-12 | 0.00; 0.006; 1E+00 | -0.022; 0.006; 2E-4 | 0.012; 0.007; 8E-2 | -0.007; 0.013; 6E-1 | 0.057; 0.009; 1E-10 |

**Supplementary table. S5. Fine mapping results for the *PKD2L1* locus.** This table presents the top 50 variants within the fine-mapped region, ranked by Posterior Inclusion Probability (PIP). For each variant, the following information is provided: rsID (variant identifier), CHR (chromosome), BP (base-pair position, build 37), EA (effect allele), OA (other allele), EAF (effect allele frequency in the European population), BETA (effect size), SE (standard error), N (sample size), and PIP (the probability that the variant is causal). The “Credible set” column indicates whether the variant is included in the 95% credible set.

| **Phenotype** | **rsID** | **CHR** | **BP** | **EA** | **OA** | **EAF** | **BETA** | **SE** | **P value** | **N** | **PIP** | **Credible set** |
| --- | --- | --- | --- | --- | --- | --- | --- | --- | --- | --- | --- | --- |
| SAT fSFA | rs603424 | 10 | 102075479 | A | G | 0.17 | 0.386 | 0.011 | 4.E-293 | 28402 | 1 | 1 |
| SAT fSFA | rs111627837 | 10 | 102062037 | CACACAT | C | 0.38 | -0.051 | 0.008 | 1.E-09 | 28402 | 0.4744254 | 1 |
| SAT fSFA | rs112090154 | 10 | 102125170 | T | A | 0.05 | -0.135 | 0.019 | 4.E-13 | 28402 | 0.2860456 | 1 |
| SAT fSFA | rs78663664 | 10 | 102100890 | C | G | 0.04 | -0.137 | 0.019 | 1.E-12 | 28402 | 0.2767839 | 1 |
| SAT fSFA | rs11190609 | 10 | 102307921 | G | A | 0.05 | -0.123 | 0.018 | 6.E-12 | 28402 | 0.1919363 | 1 |
| SAT fSFA | rs12360462 | 10 | 102063560 | G | A | 0.40 | -0.051 | 0.008 | 3.E-10 | 28402 | 0.189419 | 1 |
| SAT fSFA | rs11190469 | 10 | 102065572 | C | T | 0.40 | -0.051 | 0.008 | 3.E-10 | 28402 | 0.1711066 | 1 |
| SAT fSFA | rs3961637 | 10 | 102099802 | A | C | 0.38 | 0.107 | 0.008 | 3.E-39 | 28402 | 0.1079075 | 1 |
| SAT fSFA | rs11190623 | 10 | 102377529 | A | G | 0.05 | -0.121 | 0.018 | 2.E-11 | 28402 | 0.1045722 | 1 |
| SAT fSFA | rs683854 | 10 | 102099093 | A | C | 0.62 | -0.107 | 0.008 | 5.E-39 | 28402 | 0.0930474 | 1 |
| SAT fSFA | rs2305384 | 10 | 102056218 | A | G | 0.40 | -0.051 | 0.008 | 2.E-10 | 28402 | 0.0599012 | 1 |
| SAT fSFA | rs11190532 | 10 | 102189169 | C | T | 0.05 | -0.124 | 0.018 | 1.E-11 | 28402 | 0.057685 | 1 |
| SAT fSFA | rs7069475 | 10 | 102101280 | T | C | 0.37 | 0.106 | 0.008 | 4.E-38 | 28402 | 0.0570055 | 1 |
| SAT fSFA | rs4113432 | 10 | 102099009 | G | C | 0.38 | 0.105 | 0.008 | 3.E-37 | 28402 | 0.0555126 | 1 |
| SAT fSFA | rs59066955 | 10 | 102103529 | G | A | 0.37 | 0.104 | 0.008 | 4.E-37 | 28402 | 0.049938 | 1 |
| SAT fSFA | rs11190478 | 10 | 102102132 | C | G | 0.37 | 0.105 | 0.008 | 4.E-37 | 28402 | 0.0486091 | 1 |
| SAT fSFA | rs56014906 | 10 | 102103508 | G | A | 0.37 | 0.104 | 0.008 | 4.E-37 | 28402 | 0.0481035 | 1 |
| SAT fSFA | rs17669878 | 10 | 102099573 | C | G | 0.38 | 0.105 | 0.008 | 3.E-37 | 28402 | 0.0474225 | 1 |
| SAT fSFA | rs7071241 | 10 | 102102964 | C | T | 0.37 | 0.104 | 0.008 | 5.E-37 | 28402 | 0.0473119 | 1 |
| SAT fSFA | rs2278841 | 10 | 102056970 | G | A | 0.40 | -0.051 | 0.008 | 2.E-10 | 28402 | 0.0430635 | 1 |
| SAT fSFA | rs12261282 | 10 | 102097006 | T | G | 0.36 | 0.111 | 0.008 | 7.E-41 | 28402 | 0.0410257 | 1 |
| SAT fSFA | rs735877 | 10 | 102104521 | T | C | 0.37 | 0.104 | 0.008 | 1.E-36 | 28402 | 0.0397514 | 1 |
| SAT fSFA | rs7894688 | 10 | 102101166 | T | C | 0.32 | 0.072 | 0.009 | 4.E-17 | 28402 | 0.035706 | 1 |
| SAT fSFA | rs2278839 | 10 | 102057005 | A | G | 0.40 | -0.051 | 0.008 | 2.E-10 | 28402 | 0.0330369 | 1 |
| SAT fSFA | rs10883461 | 10 | 102095069 | T | C | 0.36 | 0.111 | 0.008 | 1.E-40 | 28402 | 0.0328516 | 1 |
| SAT fSFA | rs7086846 | 10 | 102102612 | A | G | 0.37 | 0.103 | 0.008 | 4.E-36 | 28402 | 0.0301084 | 1 |
| SAT fSFA | rs55782832 | 10 | 102097686 | A | T | 0.36 | 0.110 | 0.008 | 8.E-40 | 28402 | 0.0267135 | 1 |
| SAT fSFA | rs2275657 | 10 | 102106298 | C | G | 0.37 | 0.102 | 0.008 | 1.E-35 | 28402 | 0.0246029 | 1 |
| SAT fSFA | rs2275656 | 10 | 102106205 | C | G | 0.37 | 0.102 | 0.008 | 1.E-35 | 28402 | 0.0238541 | 1 |
| SAT fSFA | rs2148204 | 10 | 102096354 | A | C | 0.36 | 0.110 | 0.008 | 1.E-39 | 28402 | 0.022396 | 1 |
| SAT fSFA | rs10883462 | 10 | 102095122 | C | A | 0.37 | 0.109 | 0.008 | 3.E-39 | 28402 | 0.0221471 | 1 |
| SAT fSFA | rs371695045 | 10 | 102108346 | GAC | G | 0.39 | 0.100 | 0.008 | 3.E-35 | 28402 | 0.0191773 | 1 |
| SAT fSFA | rs2278840 | 10 | 102056972 | T | G | 0.39 | -0.052 | 0.009 | 1.E-09 | 28402 | 0.0180895 | 0 |
| SAT fSFA | rs1502593 | 10 | 102109202 | A | G | 0.38 | 0.100 | 0.008 | 7.E-35 | 28402 | 0.0161525 | 1 |
| SAT fSFA | rs55805447 | 10 | 102100472 | T | C | 0.26 | 0.086 | 0.009 | 2.E-21 | 28402 | 0.0156399 | 1 |
| SAT fSFA | rs3793770 | 10 | 102116914 | T | G | 0.35 | 0.104 | 0.008 | 7.E-36 | 28402 | 0.0118789 | 1 |
| SAT fSFA | rs11598792 | 10 | 101545338 | G | A | 0.08 | -0.082 | 0.015 | 2.E-08 | 28402 | 0.0095126 | 2 |
| SAT fSFA | rs2094181 | 10 | 102149039 | A | G | 0.30 | 0.072 | 0.009 | 3.E-16 | 28402 | 0.0085786 | 1 |
| SAT fSFA | rs75483041 | 10 | 101673459 | C | T | 0.08 | -0.082 | 0.014 | 1.E-08 | 28402 | 0.0083882 | 2 |
| SAT fSFA | rs11190495 | 10 | 102137133 | A | G | 0.25 | 0.082 | 0.009 | 5.E-19 | 28402 | 0.0068893 | 1 |
| SAT fSFA | rs397821863 | 10 | 102062450 | C | CAA | 0.44 | -0.071 | 0.008 | 3.E-18 | 28402 | 0.0056443 | 0 |
| SAT fSFA | rs200334162 | 10 | 102062453 | C | A | 0.44 | -0.071 | 0.008 | 3.E-18 | 28402 | 0.0056443 | 0 |
| SAT fSFA | rs77824198 | 10 | 102071181 | G | A | 0.06 | -0.146 | 0.017 | 1.E-18 | 28402 | 0.0050355 | 1 |
| SAT fSFA | rs112285384 | 10 | 102103037 | T | C | 0.12 | -0.073 | 0.012 | 1.E-09 | 28402 | 0.0044571 | 1 |
| SAT fSFA | rs75491399 | 10 | 102162665 | C | A | 0.08 | -0.083 | 0.015 | 2.E-08 | 28402 | 0.0040958 | 2 |
| SAT fSFA | rs75090039 | 10 | 102196831 | T | C | 0.14 | -0.064 | 0.012 | 4.E-08 | 28402 | 0.0038511 | 2 |
| SAT fSFA | rs79350316 | 10 | 102493835 | T | C | 0.11 | 0.092 | 0.013 | 5.E-13 | 28402 | 0.0035498 | 1 |
| SAT fSFA | rs74942995 | 10 | 102105630 | C | T | 0.13 | -0.071 | 0.012 | 2.E-09 | 28402 | 0.0034555 | 0 |
| SAT fSFA | rs79672037 | 10 | 102203541 | G | C | 0.11 | -0.070 | 0.013 | 3.E-08 | 28402 | 0.0027143 | 0 |
| SAT fSFA | rs76731979 | 10 | 101953275 | G | C | 0.06 | -0.138 | 0.017 | 5.E-17 | 28402 | 0.0026435 | 1 |

**Supplementary table S6. Functional annotation of loci associated with adipose tissue fatty acid composition.** FUNC: functional annotation of the variant; CADD: Combined Annotation Dependent Depletion score (>12.37 indicate high probability of deleteriousness); RDB: RegulomeDB score, lower scores indicate stronger evidence of regulatory function; GWAS P: P value reported by GWAS; ciMap: evidence of chromatin interactions; cis eQTL tissue: the tissue where the SNP is significantly associated with gene expression, P value: multiple test adjusted p value; direction: the direction of gene expression association for the effect allele; cis pQTL: the plasma protein levels within 1Mb of the variant.

| **rsID** | **Effect allele** | **FUNC** | **CHR** | **CADD** | **RDB** | **GWAS P** | **ciMap** | **Cis eQTL tissue, gene, direction, P value** | **Cis pQTL protein, P value** |
| --- | --- | --- | --- | --- | --- | --- | --- | --- | --- |
| rs603424 | G | Intronic | 10 | 4.567 | 3a | 2 E-126 | No | Adipose Visceral Omentum, *SCD*, +, 2x10^-6^  Adipose Subcutaneous, *SCD*, +, 2x10^-6^  Brain Frontal Cortex, *PKD2L1*, -, 1x10^-6^ | DNMBP_Q6XZF7_OID20956_v1_Neurology, 5x10^-4^ |
| rs59186169 | A | Intergenic | 7 | 0.12 | 2b | 2 E-11 | No | NA | SHH_Q15465_OID30516_v1_Inflammation_II, 6x10^-3^ |
| rs73221948 | G | Intergenic | 8 | 7.9 | NA | 4 E-11 | No | NA | NA |
| rs67261871 | T | Intergenic | 3 | 14.8 | 6 | 1 E-10 | Yes | Adipose Subcutaneous, *TIPARP*, +, 2x10^-3^  Adipose Visceral Omentum, *TIPARP*, +, 4x10^-2^ |  |
| rs10260148 | C | intergenic | 7 | 2.9 | 7 | 2 E-12 | No | NA | CPA2_P48052_OID21060_v1_Neurology, 4x10^-10^  CPA4_Q9UI42_OID30606_v1_Inflammation_II, 4x10^-14^ |
| rs660745 | T | Intronic | 19 | 2.9 | 7 | 2 E-8 | Yes | Brain Hypothalamus, *NTN5*, -, 2x10^-4^  Brain Hypothalamus, *MAMSTR*, -, 1x10^-7^ | GYS1_P13807_OID20097_v1_Cardiometabolic, 3x10^-3^  CA11_O75493_OID21195_v1_Oncology, 5x10^-5^  FGF21_Q9NSA1_OID21402_v1_Oncology, 3x10^-2^  HSD17B14_Q9BPX1_OID31098_v1_Neurology_II, 3x10^-4^  FUT1_P19526_OID31295_v1_Oncology_II, 5x10^-3^ |

**Supplementary table S7. Colocalization analysis of genetic loci associated with subcutaneous adipose tissue saturated fatty acids (fSFA).** nSNP: number of independent variants analysed within the genomic region; Trait; cardiometabolic traits analysed based on their association with the genetic loci; rsID: RS identifier of the variant driving the colocalization signal; CHR: chromosome; PP.H0: posterior probability (PP) that neither trait is associated with the genetic locus, PP.H1: PP that the variant is associated only with fSFA, PP.H2: PP that the variant is associated only with the second trait (e.g., a cardiometabolic trait), PP.H3: PP that distinct causal variants influence both fSFA and the second trait (suggesting independent genetic regulation), PP.H4: PP that the same locus is associated with both traits (evidence for colocalization), SNP.PP.H4: PP that the specific SNP is the shared causal variant for both traits.

| **nSNP** | **Trait** | **rsID** | **CHR** | **Locus** | **PP.H0** | **PP.H1** | **PP.H2** | **PP.H3** | **PP.H4** | **SNP.PP.H4** |
| --- | --- | --- | --- | --- | --- | --- | --- | --- | --- | --- |
| 195 | Hypertension | rs603424 | 10 | *PKD2L1* | 1.4E-287 | 1.5E-03 | 9.1E-288 | 4.5E-06 | 1.0E+00 | 100% |
| 132 | Cholithiasis | rs603424 | 10 | *PKD2L1* | 7.7E-288 | 9.4E-04 | 2.6E-287 | 2.2E-03 | 1.0E+00 | 100% |
| 245 | Hypertension | rs12154627 | 7 | *KLF14* | 5.7E-21 | 1.5E-14 | 3.9E-10 | 2.7E-05 | 1.0E+00 | 100% |
| 217 | Type 2 diabetes | rs12154627 | 7 | *KLF14* | 5.6E-16 | 1.5E-09 | 7.1E-09 | 1.8E-02 | 9.8E-01 | 94% |
| 339 | Hypertension | rs479486 | 19 | *RASIP1* | 3.2E-05 | 1.2E-05 | 2.6E-02 | 8.3E-03 | 9.7E-01 | 98% |
| 294 | Cholithiasis | rs11671705 | 19 | *SULT2B1* | 2.8E-01 | 9.8E-02 | 2.8E-01 | 9.8E-02 | 2.5E-01 | 40% |

**Supplementary table S8. Gene–diet interaction analysis for rs603424.** This table presents the results of interaction analyses between the rs603424 genotype and dietary fatty acid intake on cardiometabolic disease outcomes. Interaction terms were tested using logistic regression models adjusted for age, sex, and ethnicity

| **Diet** | **Outcome** | **Beta** | **Standard error** | **z** | **p-value** |
| --- | --- | --- | --- | --- | --- |
| Monounsaturated fatty acids | Hypertension | 0.002158943 | 0.002421792 | 0.891465299 | 0.3726796 |
| Monounsaturated fatty acids | Type 2 diabetes | -0.001649852 | 0.004355675 | -0.378782233 | 0.704849582 |
| Monounsaturated fatty acids | Cardiovascular disease | 0.00097824 | 0.003898355 | 0.250936585 | 0.801863138 |
| Polyunsaturated fatty acids | Hypertension | 0.003393469 | 0.003474195 | 0.976764255 | 0.328685876 |
| Polyunsaturated fatty acids | Type 2 diabetes | 0.000108006 | 0.00632143 | 0.017085742 | 0.986368213 |
| Polyunsaturated fatty acids | Cardiovascular disease | 0.001786306 | 0.005549925 | 0.321861263 | 0.747557801 |
| Saturated fatty acids | Hypertension | 0.002945395 | 0.002243074 | 1.313106617 | 0.189147025 |
| Saturated fatty acids | Type 2 diabetes | 0.002335727 | 0.004231413 | 0.551996895 | 0.580950481 |
| Saturated fatty acids | Cardiovascular disease | 0.00732924 | 0.003593469 | 2.039600284 | 0.041390154 |


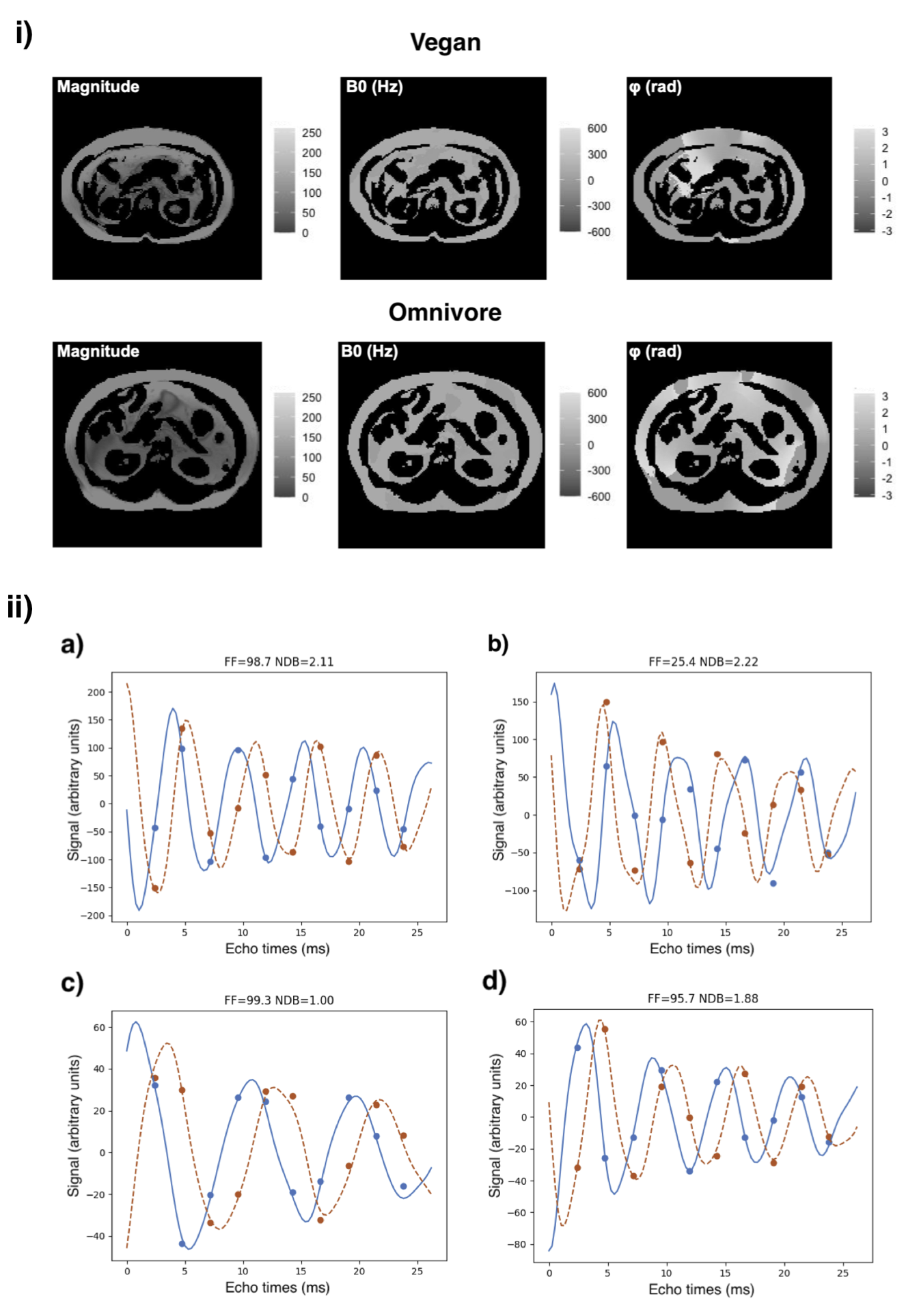


**Supplementary Figure S1.**  **i)** Maps of magnitude, field inhomogeneity (B_0_) and phase (φ) of the vegan and omnivore participants (2). **ii)** Examples of fitted complex-valued data for representative voxels in the omnivore participant from Figure 1. The blue points correspond to the real values and the red points correspond to the imaginary values. The estimated models are solid lines for the real values and dashed lines for the imaginary values. Voxels were taken from (a) the VAT, (b) the ASAT, (c) the potential artifactual variation on the left-hand side of the ASAT, and (d) the potential artifactual variation on the right-hand side of the ASAT.


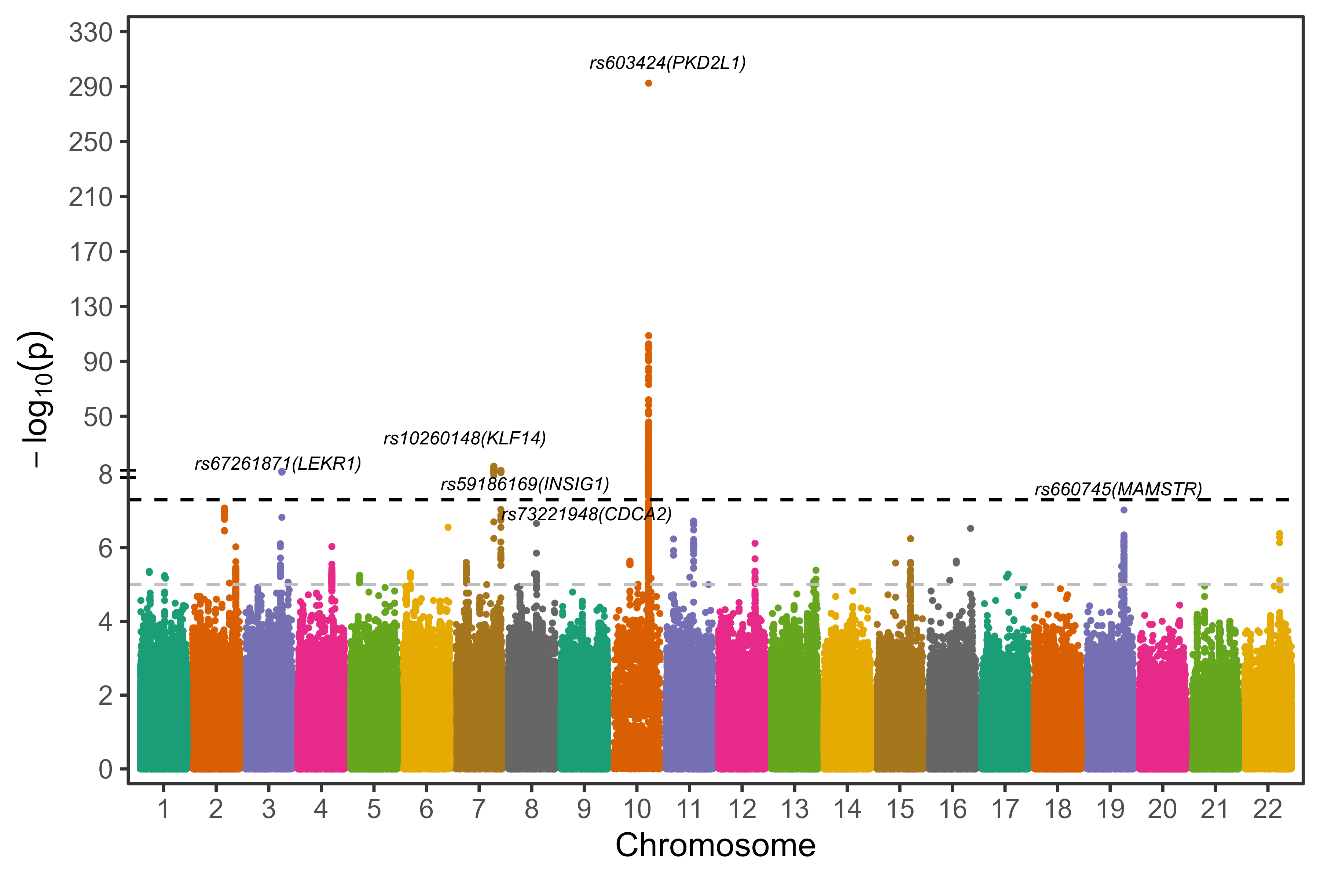


**Supplementary Figure S2. Genetic loci associated with SAT and VAT fatty acids composition.** The Manhattan plot shows the six loci associated with SAT and VAT fatty acids composition and their nearest genes. The x-axis represents the chromosomes 1-22, and the y-axis shows -log10 pvalue for all genetic variants. The black dashed line is the genome wide significance threshold of 5 x 10^-8^.


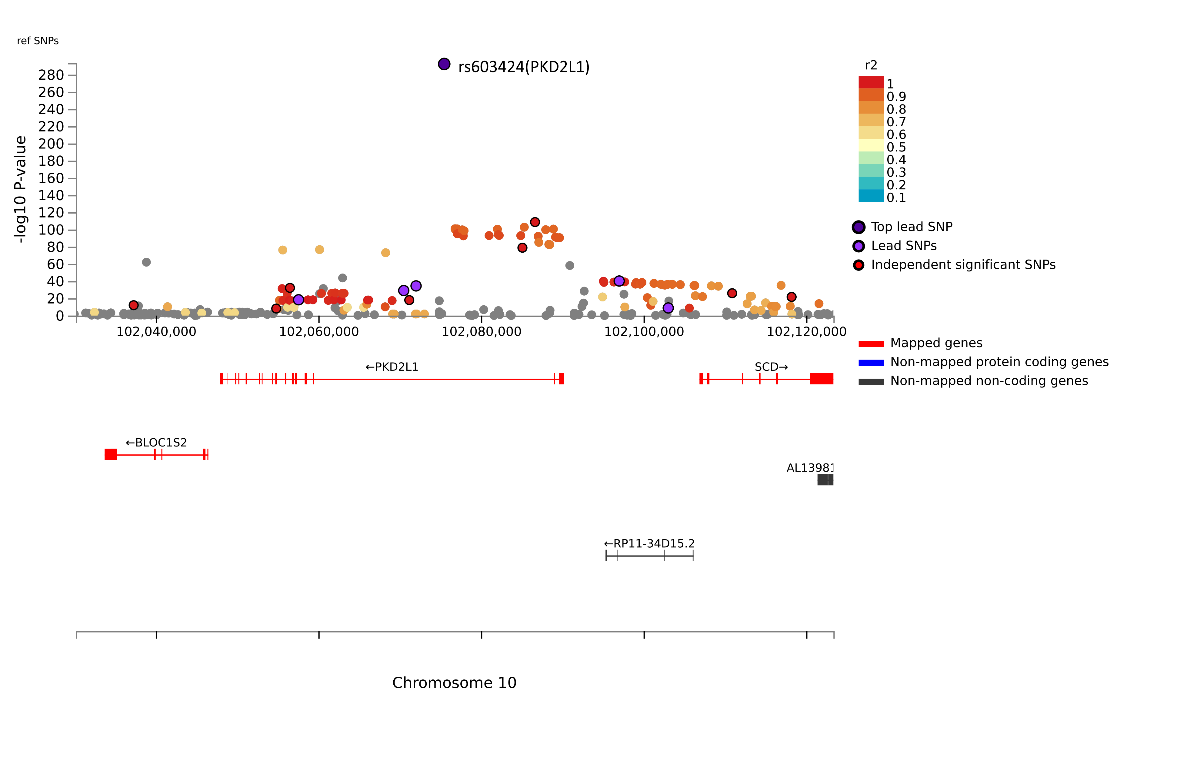


**Supplementary figure S3. Regional association plot for rs603424(*PKD2L1)* locus on SAT SFA chromosome 10 from FUMA.** The locus zoom centred on rs603424 which identified as potential causal variant for regulating SAT and VAT fatty acids composition. The x-axis is genomic coordinates on chromosome 10 (Genomic build 37), the y-axis is -log10(pvalue) for the SNPs association with SAT SFA and the coloured points represents the variants that in **linkage disequilibrium based on R^2^** with the lead SNP (rs603424), using 1000 Genomes European population reference.


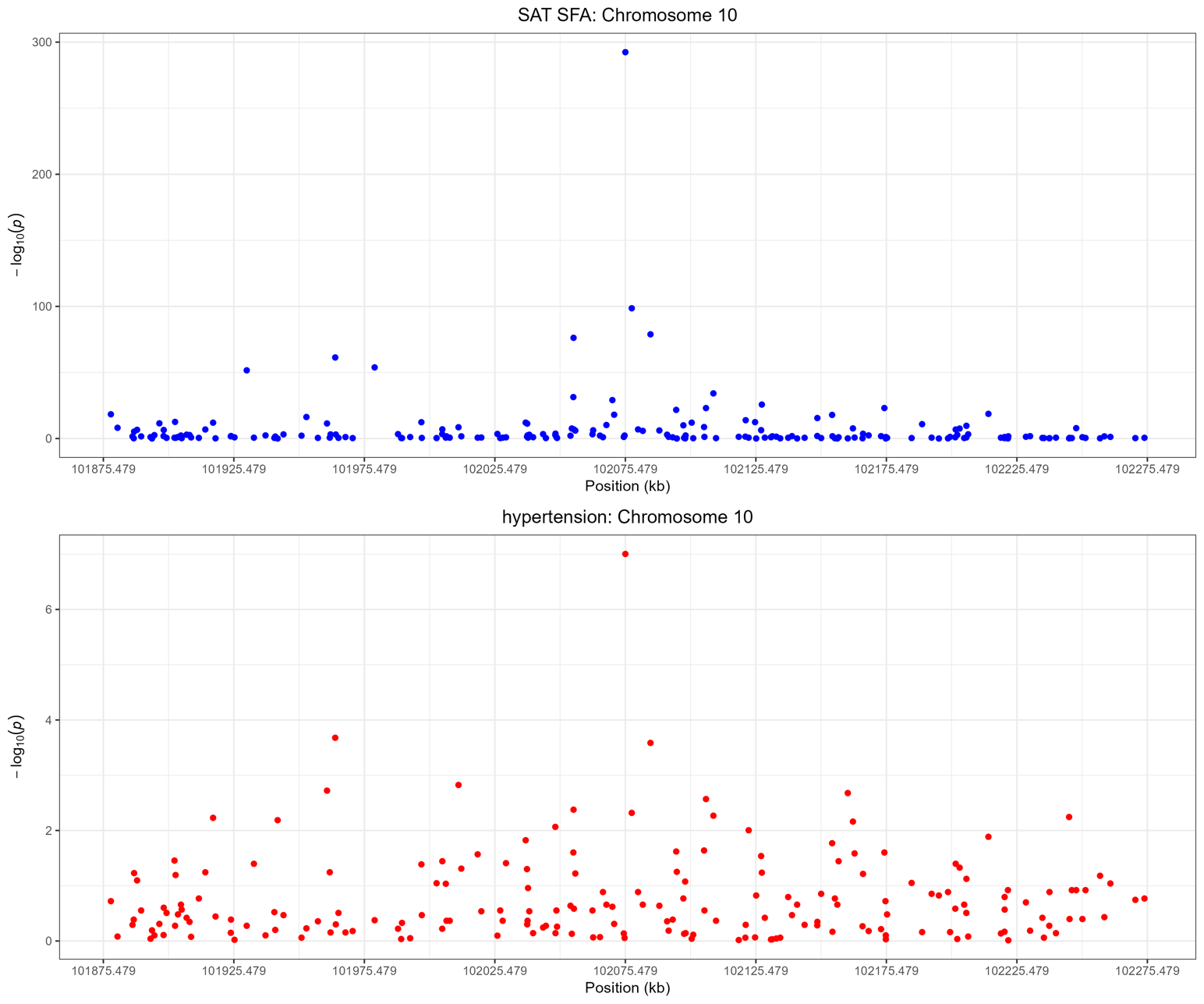


**Supplementary Figure S4. Regional association plots for SAT fSFA and hypertension at the *PKD2L1* locus on chromosome 10.** The upper panel (blue) shows the −log₁₀(p) values for associations between genetic variants and saturated fatty acid levels in subcutaneous adipose tissue (SAT fSFA). The lower panel (red) displays associations with hypertension. The x-axis denotes genomic coordinates (kilobases, kb), and the y-axis indicates −log₁₀(p), reflecting the strength of association for each variant. Overlapping association peaks suggest potential colocalisation of causal signals between adipose tissue fatty acid composition and hypertension.


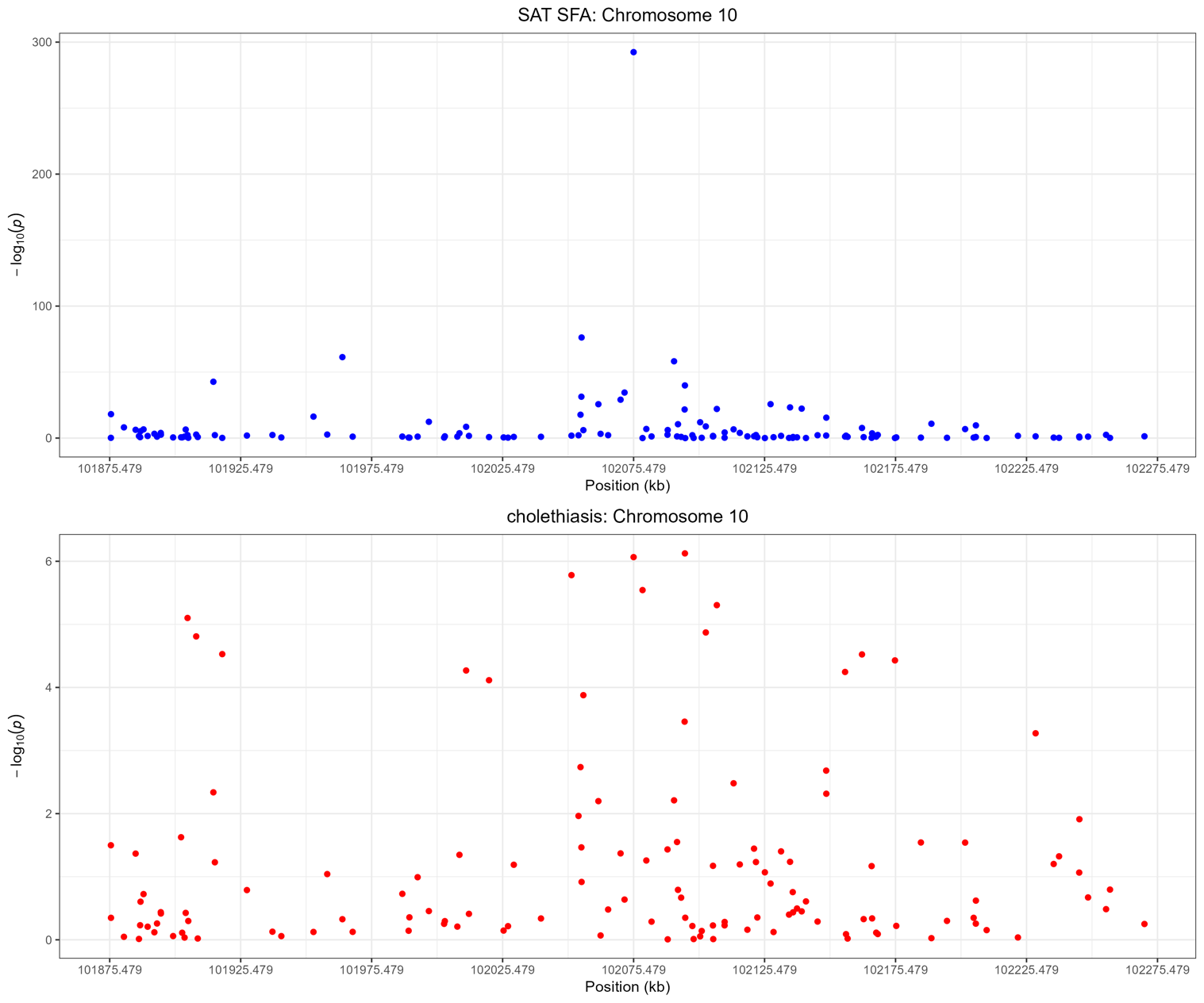


**Supplementary Figure S5. Regional association plots for SAT fSFA and cholithiasis at the *PKD2L1* locus on chromosome 10.** The upper panel (blue) shows the −log₁₀(p) values for associations between genetic variants and saturated fatty acid levels in subcutaneous adipose tissue (SAT fSFA). The lower panel (red) displays associations with cholithiasis. The x-axis denotes genomic coordinates (kilobases, kb), and the y-axis indicates −log₁₀(p), reflecting the strength of association for each variant. Overlapping association peaks suggest potential colocalisation of causal signals between adipose tissue fatty acid composition and hypertension.


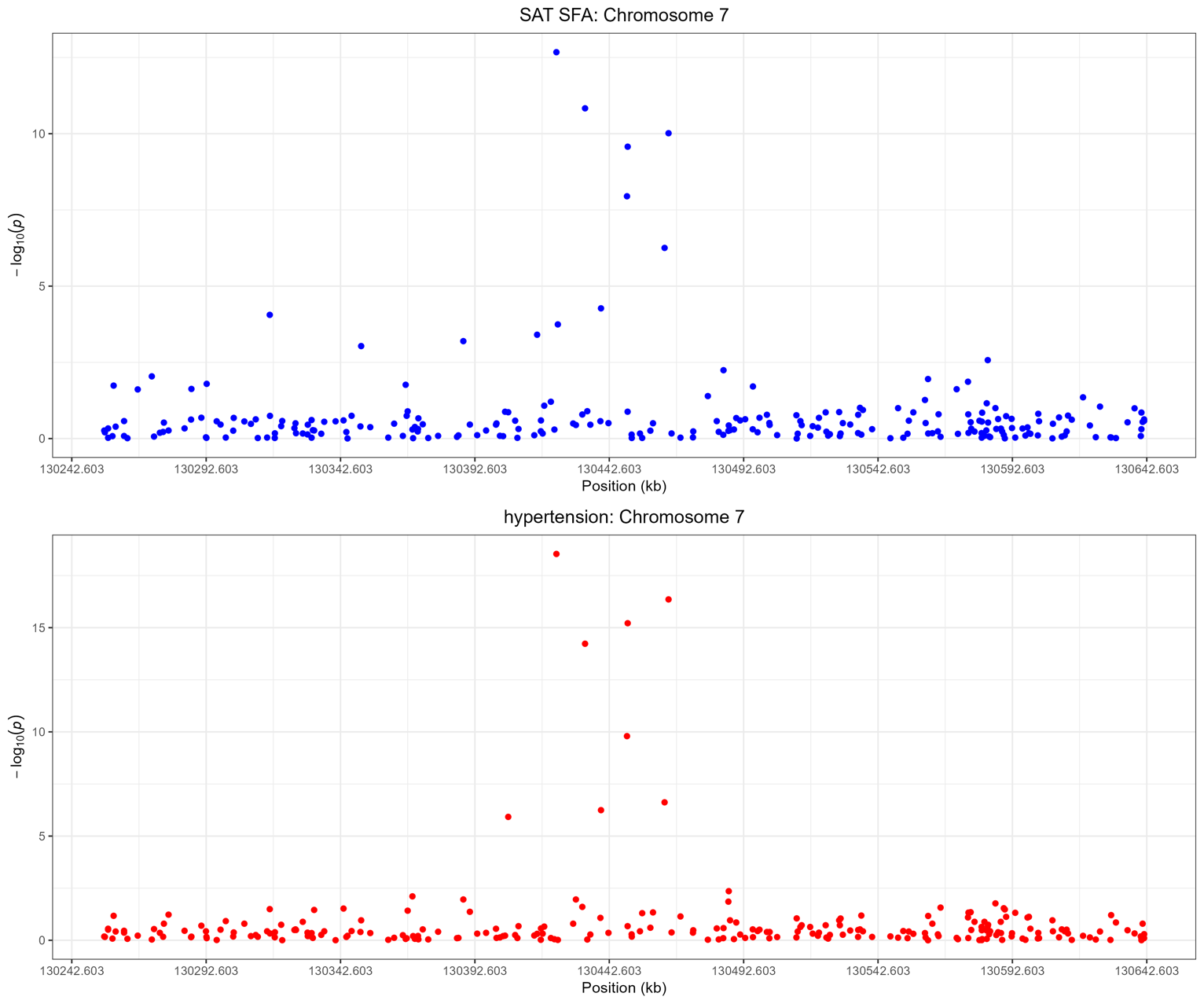


**Supplementary Figure S6. Regional association plots for SAT fSFA and hypertension at the *KLF14* locus on chromosome 7.** The upper panel (blue) shows the −log₁₀(p) values for associations between genetic variants and saturated fatty acid levels in subcutaneous adipose tissue (SAT fSFA). The lower panel (red) displays associations with hypertension. The x-axis denotes genomic coordinates (kilobases, kb), and the y-axis indicates −log₁₀(p), reflecting the strength of association for each variant. Overlapping association peaks suggest potential colocalisation of causal signals between adipose tissue fatty acid composition and hypertension.


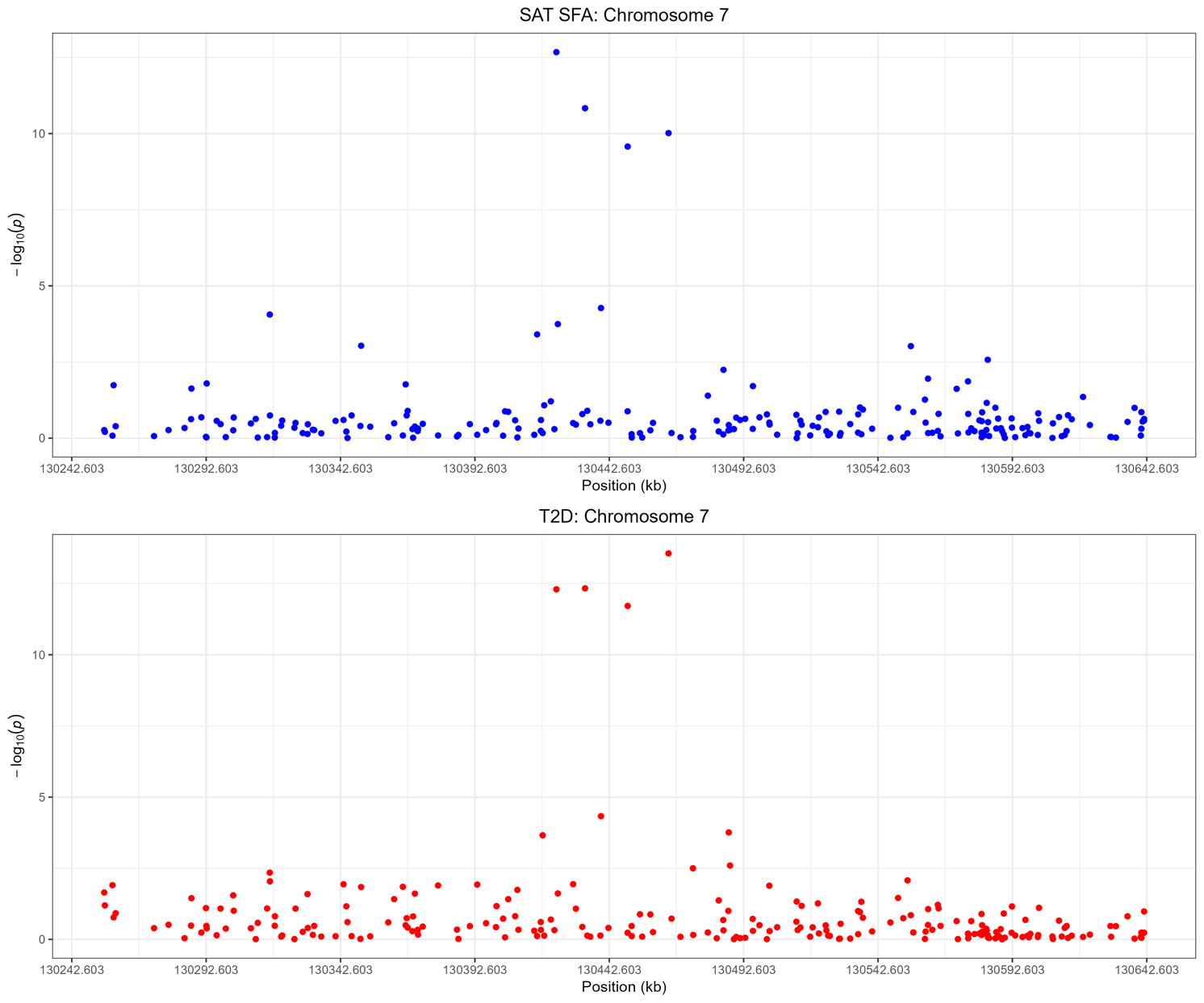


**Supplementary Figure S7. Regional association plots for SAT fSFA and type 2 diabetes at the *KLF14* locus on chromosome 7.** The upper panel (blue) shows the −log₁₀(p) values for associations between genetic variants and saturated fatty acid levels in subcutaneous adipose tissue (SAT fSFA). The lower panel (red) displays associations with type 2 diabetes. The x-axis denotes genomic coordinates (kilobases, kb), and the y-axis indicates −log₁₀(p), reflecting the strength of association for each variant. Overlapping association peaks suggest potential colocalisation of causal signals between adipose tissue fatty acid composition and hypertension.


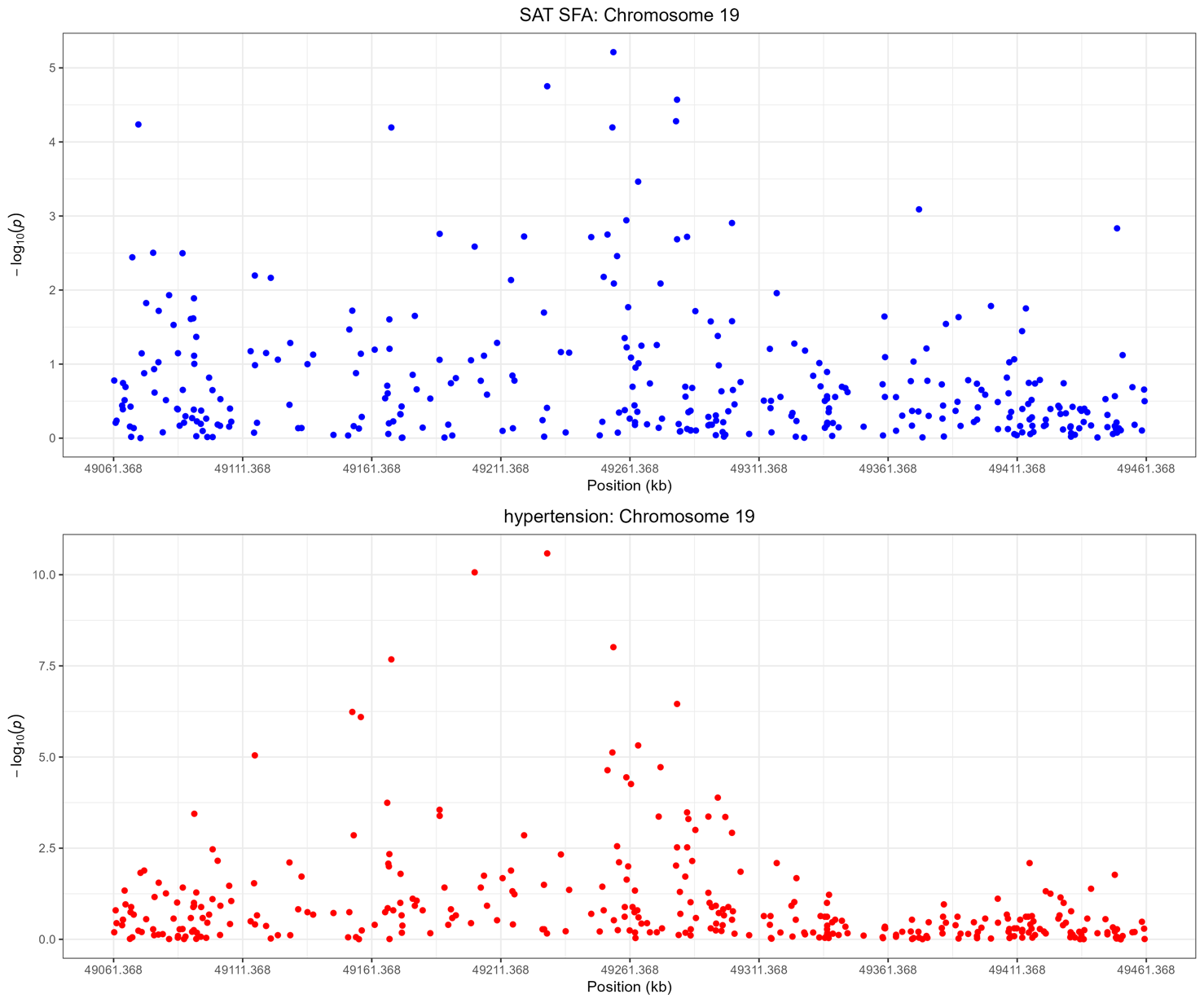


**Supplementary Figure S8. Regional association plots for SAT fSFA and hypertension at the *MAMSTR* locus on chromosome 19.** The upper panel (blue) shows the −log₁₀(p) values for associations between genetic variants and saturated fatty acid levels in subcutaneous adipose tissue (SAT fSFA). The lower panel (red) displays associations with hypertension. The x-axis denotes genomic coordinates (kilobases, kb), and the y-axis indicates −log₁₀(p), reflecting the strength of association for each variant. Overlapping association peaks suggest potential colocalisation of causal signals between adipose tissue fatty acid composition and hypertension.


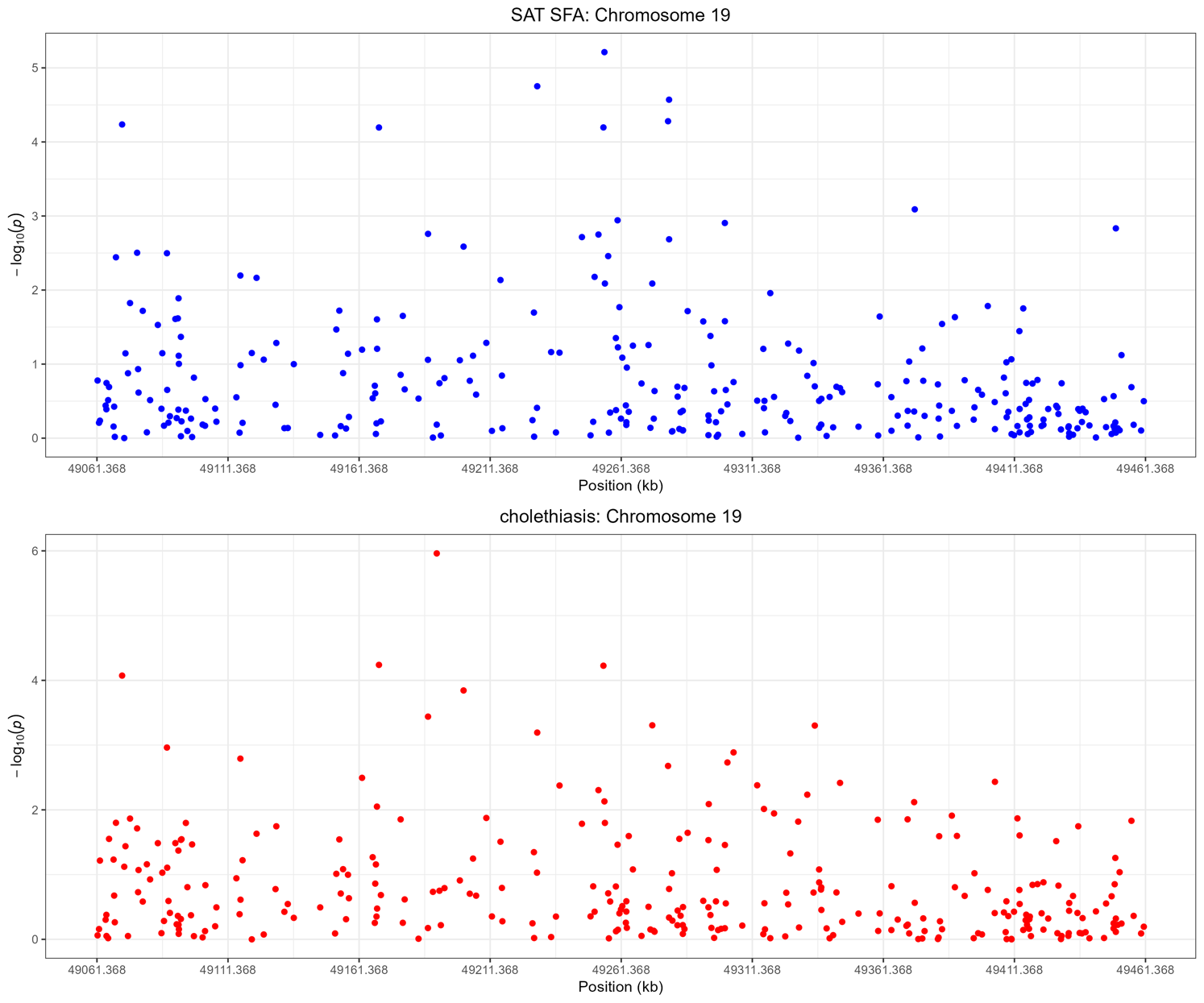


**Supplementary Figure S9. Regional association plots for SAT fSFA and cholelithiasis at the *MAMSTR* locus on chromosome 19.** The upper panel (blue) shows the −log₁₀(p) values for associations between genetic variants and saturated fatty acid levels in subcutaneous adipose tissue (SAT fSFA). The lower panel (red) displays associations with cholethiasis. The x-axis denotes genomic coordinates (kilobases, kb), and the y-axis indicates −log₁₀(p), reflecting the strength of association for each variant. Overlapping association peaks suggest potential colocalisation of causal signals between adipose tissue fatty acid composition and hypertension.

**References**

1. Bydder M, Girard O, Hamilton G. Mapping the double bonds in triglycerides. Magnetic Resonance Imaging. 2011;29(8):1041-6.

2. Thanaj M, Basty N, Whitcher B*, et al.* M RI assessment of adipose tissue fatty acid composition in the UK Biobank and its association with diet and disease. Obesity. 2024;32(9):1699-708.
